# A Telomere-to-telomere Diploid Reference Genome and Centromere Structure of the Chinese Quartet

**DOI:** 10.1093/gpbjnl/qzaf118

**Published:** 2025-11-26

**Authors:** Bo Wang, Peng Jia, Stephen J Bush, Xia Wang, Yi Yang, Yu Zhang, Shijie Wan, Xiaofei Yang, Pengyu Zhang, Yuanting Zheng, Leming Shi, Lianhua Dong, Kai Ye

**Affiliations:** School of Automation Science and Engineering, Faculty of Electronic and Information Engineering, Xi’an Jiaotong University, Xi’an 710049, China; MOE Key Lab for Intelligent Networks & Networks Security, Faculty of Electronic and Information Engineering, Xi’an Jiaotong University, Xi’an 710049, China; Department of Gynecology and Obstetrics, Center for Mathematical Medical, The First Affiliated Hospital of Xi’an Jiaotong University, Xi’an 710061, China; School of Automation Science and Engineering, Faculty of Electronic and Information Engineering, Xi’an Jiaotong University, Xi’an 710049, China; Center for Advanced Measurement of Science, National Institute of Metrology, Beijing 100029, China; Center for Advanced Measurement of Science, National Institute of Metrology, Beijing 100029, China; Shenzhen Institute for Technology Innovation, National Institute of Metrology, Shenzhen 518107, China; Center for Advanced Measurement of Science, National Institute of Metrology, Beijing 100029, China; School of Computer Science and Technology, Faculty of Electronic and Information Engineering, Xi’an Jiaotong University, Xi’an 710049, China; School of Computer Science and Technology, Faculty of Electronic and Information Engineering, Xi’an Jiaotong University, Xi’an 710049, China; School of Automation Science and Engineering, Faculty of Electronic and Information Engineering, Xi’an Jiaotong University, Xi’an 710049, China; State Key Laboratory of Genetic Engineering, Human Phenome Institute and School of Life Sciences, Fudan University, Shanghai 200438, China; State Key Laboratory of Genetic Engineering, Human Phenome Institute and School of Life Sciences, Fudan University, Shanghai 200438, China; Center for Advanced Measurement of Science, National Institute of Metrology, Beijing 100029, China; Shenzhen Institute for Technology Innovation, National Institute of Metrology, Shenzhen 518107, China; School of Automation Science and Engineering, Faculty of Electronic and Information Engineering, Xi’an Jiaotong University, Xi’an 710049, China; MOE Key Lab for Intelligent Networks & Networks Security, Faculty of Electronic and Information Engineering, Xi’an Jiaotong University, Xi’an 710049, China; Department of Gynecology and Obstetrics, Center for Mathematical Medical, The First Affiliated Hospital of Xi’an Jiaotong University, Xi’an 710061, China; Faculty of Science, Leiden University, 2311 EZ, Leiden, The Netherlands

**Keywords:** Genome assembly, Complex genomic region, Centromere, Higher-order repeat, Monozygotic twins

## Abstract

Recent advances in sequencing technologies have enabled the complete assembly of human genomes from telomere to telomere (T2T), resolving previously inaccessible regions such as centromeres and segmental duplications. Here, we present an updated, higher-quality, haplotype-phased T2T assembly of the Chinese Quartet (T2T-CQ), a family cohort comprising monozygotic twins and their parents, generated using high-coverage Oxford Nanopore Technologies (ONT) ultralong and PacBio high-fidelity (HiFi) sequencing. The T2T-CQ assembly serves as a crucial reference genome for integrating publicly available multi-omics data and advances the utility of the Quartet reference materials. The T2T-CQ assembly scores highly on multiple metrics of continuity and completeness, with Genome Continuity Inspector (GCI) scores of 77.76 (maternal) and 76.41 (paternal), 21-mer quality values (QVs) > 66, and Clipping Reveals Assembly Quality (CRAQ) scores > 99.6 for both haplotypes, enabling complete annotation of centromeric regions. Within these regions, we identified novel 13-mer higher-order repeat patterns on chromosome 17 which exhibited a monophyletic origin and emerged approximately 230 thousand years ago. Overall, this work establishes an essential genomic resource for the Han Chinese population and advances the development of a T2T pan-Chinese reference genome, which will significantly enable future investigations both into population-specific structural variants and the evolutionary dynamics of centromeres.

## Graphical Abstract

**Figure:**
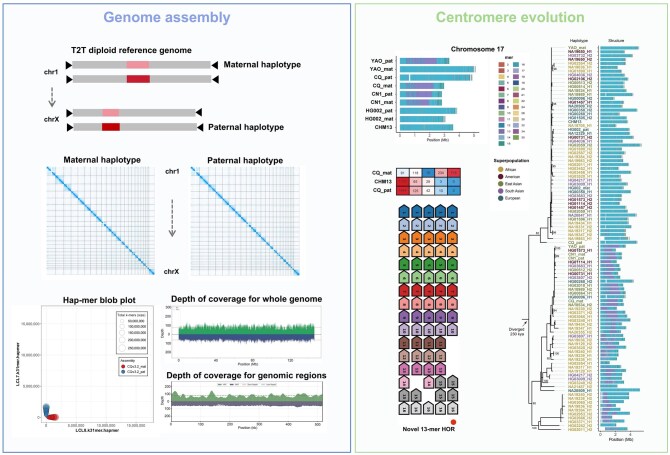

## Introduction

The completion of the first telomere-to-telomere (T2T) human genome assembly, the primarily European T2T-CHM13 [1], was a landmark achievement in genomics, providing an updated reference for exploring the full complexity of the human genome, including previously unresolved regions such as centromeres, telomeres, rDNAs, and segmental duplications. This breakthrough was facilitated by advancements in long-read sequencing technologies, specifically Oxford Nanopore Technologies (ONT) and PacBio high-fidelity (HiFi) platforms, which enabled comprehensive resolution of highly repetitive and structurally complex genomic regions, contributing to a more complete understanding of human genetic variation [2–4]. Several high-quality Chinese reference genomes, including YH2.0 [5], HX1 [6], NH1.0 [7], Han1 [8], and HJ [9], have also been successfully assembled and released. However, these assemblies remain either partially collapsed or contain unresolved gaps, indicating room for further improvement in completeness and accuracy. Following this, and to close gaps in ethnic diversity, the T2T-CN1 [10] and T2T-YAO [11] assemblies were released, representing complete diploid genomes of Han Chinese individuals and highlighting the importance of high-quality, population-specific reference genomes to capture the full spectrum of complex genetic variation [12]. Building on these achievements, we present the T2T-CQ genome, a high-quality, haplotype-resolved T2T assembly generated from a family of Han Chinese descent [13,14]. The Chinese Quartet project (https://chinese-quartet.org/) is a suite of DNA, RNA, protein, and metabolite reference materials from B-lymphoblastoid cell lines (LCLs) from two monozygotic twin daughters (LCL5 and LCL6) and their biological parents (LCL7 and LCL8 for father and mother, respectively), extensively detailed in previous publications [13–18]. Our initial assembly of the CQ family [16], hereafter CQ v2.0, laid the groundwork for the present study in which we refine and expand it. By incorporating high-depth ultralong ONT reads (> 100 kb), we significantly enhance both the contiguity and completeness of CQ v2.0 to T2T-level quality, with both maternal and paternal haplotypes attaining 21-mer quality value (QV) over 66.

Centromeres, which are essential for chromosome segregation during cell division, remain among the most challenging regions to assemble due to their highly repetitive nature and structural complexity [2,19]. T2T-level assemblies therefore enable comprehensive annotations of centromeric regions, including the identification of higher-order repeats (HORs), which are essential for understanding centromere structure and function [3,20,21]. As such, we focus on characterizing centromeric regions in the T2T-CQ genome, identifying novel HORs and investigating their emergence and evolution. Additionally, we evaluate the performance of two state-of-the-art genome assembly tools, hifiasm and Verkko, using our twin sequencing data as a robust benchmark. With the availability of a T2T-level reference genome, the Chinese Quartet provides an invaluable model for studying centromere biology, genetic variation, and evolutionary dynamics.

## Results and discussion

### A diploid T2T genome assembly for the Chinese Quartet

**Figure 1:**
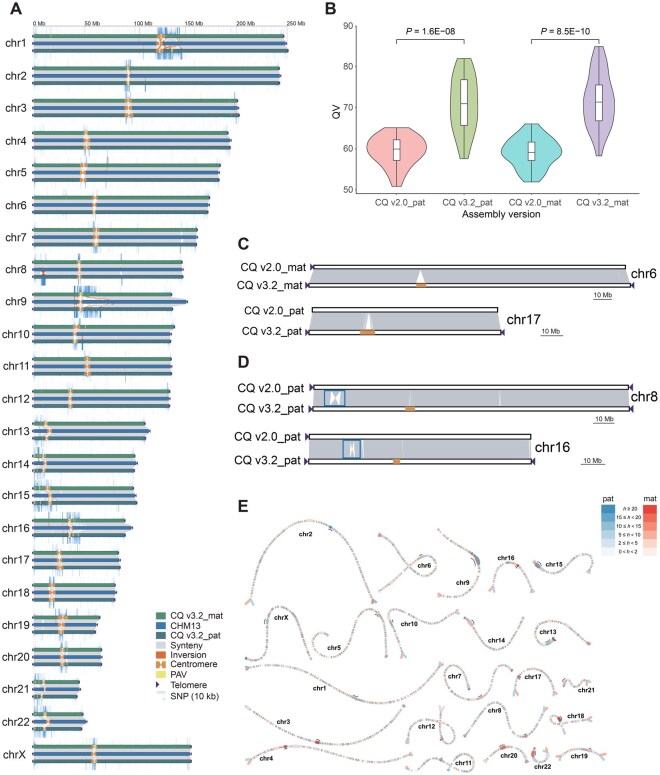
Overview of CQ v3.2 and its comparison with CQ v2.0 **A**.Comparison of the CQ v3.2 and CHM13 reference genomes. Each set of chromosomes includes the maternal haplotype of CQ v3.2 (CQ v3.2_mat, top), the paternal haplotype of CQ v3.2 (CQ v3.2_pat, bottom), and CHM13 (middle). **B**. Comparison of the QV (*k* = 21) for each chromosome of the maternal and paternal haplotypes in CQ v3.2 and CQ v2.0. Significance was tested by Wilcoxon rank-sum test. **C**. Illustration of the advantage of CQ v3.2 over CQ v2.0 in centromeric region (highlighted in orange bars) assembly. **D**. Illustration of large inversion variation (highlighted in blue boxes) between CQ v3.2 and CQ v2.0. **E**. Visualization of heterozygous regions between two haplotypes using bubbles. Regions with different heterozygous variants are displayed in distinct colors, while centromeric regions are represented by black lines. The heterozygosity rate (*h*) is calculated as the structural variant count in each 500-kb window. PAV, Presence-Absence Variation; SNP, single-nucleotide polymorphism; QV, quality value; pat, paternal; mat, maternal.

In previous work [16], we presented CQ v2.0, haplotype-resolved assemblies of the monozygotic twins. However, although these assemblies were highly contiguous, they fell short of T2T standard, with the maternal haplotype assembly (CQ v2.0_mat) containing 34 gaps, and the paternal (CQ v2.0_pat) 51 gaps (**Table 1**). Moreover, 17 and 20 telomere motifs were missing in the maternal and paternal CQ v2.0 assemblies, respectively (Table 1). In this study, we supplemented our previously used set of 10.3× ONT ultralong reads (> 100 kb) with an additional 57.3× (Table S1), aiming to assemble a T2T-level haplotype-resolved genome to serve as a new reference genome for the CQ. We constructed the initial assemblies using two state-of-the-art tools, Verkko [22,23] and hifiasm [24], in trio binning mode. As described in the Materials and methods section and illustrated in Figure S1A, the contigs from the two assembly results were merged for the maternal haplotype, leaving 9 gaps distributed across chromosomes 1, 6, 10, 11, 13, 14, 15, 21, and 22. Except for those in the short arms of acrocentric chromosomes (SAACs) 13, 14, 15, 21, and 22, gaps in the other chromosomes were filled using TGS-GapCloser [25] in conjunction with binning ONT reads and the ONT-based hifiasm assembly (Figure S1B). For the paternal haplotype, 8 gaps remained, located on chromosomes 4, 5, 13, 14, 15, 21, 22, and X, which we also filled using TGS-GapCloser. The rDNA clusters in the SAACs of both maternal and paternal haplotypes were finalized by adopting the strategy employed for T2T-CN1 [10]. Briefly, this involved predicting the rDNA copy number (CN) using droplet digital PCR (ddPCR), followed by evaluating the rDNA CN for each chromosome using the T2T-CHM13 major rDNA morphs [26]. The ddPCR results showed that, as expected, the copy ratios of the two twins were similar, with values of 276.92 and 283.73, respectively. Since we combined data from the twins (see Materials and methods), we used their average value of 280 as the rDNA CN. Following the approach described in the “rDNA analysis” section of the Materials and methods, we found that the rDNA CNs for CQ v3.2_mat and CQ v3.2_pat were 123 and 157, respectively (Table 1). The five rDNA major morphs used for genome filling correspond to the five SAACs, with the rDNA CN for each of these chromosomes in CQ v3.2_mat and CQ v3.2_pat detailed in Tables S2 and S3, respectively. We polished the assembled genome using NextPolish2 [27], ultimately obtaining T2T diploid human genome assemblies, designated as CQ v3.0_mat (maternal haplotype) and CQ v3.0_pat (paternal haplotype) (**Figure 1**A). Subsequently, we integrated the mitochondrial genome into the maternal haplotype assembly and performed structural variant (SV) error correction using a previously published pipeline [28], followed by manual validation. This refinement process identified and resolved three SVs in CQ v3.0_pat (Figure S2), whereas no SVs were detected in CQ v3.0_mat. The resulting high-quality T2T assembly was designated as the final CQ v3.2 genome. These two haplotype-resolved assemblies were further validated using high-throughput chromosome conformation capture (Hi-C) contact maps and manual inspection, which confirmed the absence of significant assembly errors (Figure S3A and B).

**Table 1:** Comparison of assembly quality among human T2T genome assemblies

Statistic	CQ v2.0_mat	CQ v2.0_pat	CQ v3.2_mat	CQ v3.2_pat	CN1 v1.0.1_mat	CN1 v1.0.1_pat	YAO v1.1_mat	YAO v1.1_pat
Assembly size (Gb)	3.00	3.00	3.03	3.03	3.04	2.94	3.02	2.92
No. of gaps	34	51	0	0	0	0	0	0
No. of telomeres	29	26	46	46	46	46	46	46
Contig N50 (Mb)	132.84	132.84	155.11	155.17	157.36	145.78	155.06	145.07
QV range among chromosomes (*k* = 21)	51.82–65.74	50.81–65.00	57.94–83.11	57.32–81.81	47.33–70.83	51.91–73.02	60.58–85.47	63.39–91.52
QV whole-genome (*k* = 21)	57.42	58.15	67.79	66.59	60.11	59.37	70.49	72.28
QV range among chromosomes (*k* = 31)	49.03–61.94	49.50–62.16	55.07–73.48	54.69–73.24	NA	NA	NA	NA
QV whole-genome (*k* = 31)	54.60	55.32	63.72	63.15	NA	NA	NA	NA
False duplication rate (%) (*k* = 21)	0.41	0.44	0.16	0.14	NA	NA	0.30	0.20
False duplication rate (%) (*k* = 31)	0.44	0.48	0.18	0.16	NA	NA	NA	NA
Haplotype switch error rate (%) (*k* = 21)	0.16	0.15	0.19	0.20	NA	NA	0.02	0.01
Haplotype switch error rate (%) (*k* = 31)	0.26	0.26	0.22	0.23	NA	NA	NA	NA
*K*-mer completeness (%; haploid) (*k* = 21)	97.83	97.90	97.95	97.95	NA	NA	NA	NA
*K*-mer completeness (%; haploid) (*k* = 31)	96.75	96.84	96.92	96.92	NA	NA	NA	NA
*K*-mer completeness (%; diploid) (*k* = 21)	99.54		99.57		NA	NA	NA	NA
*K*-mer completeness (%; diploid) (*k* = 31)	99.37		99.42		NA	NA	NA	NA
GCI (HiFi + ONT)	NA	NA	77.76	76.41	66.79	77.90	NA	NA
Chromosome composition	22 + X	22 + X	22 + X	22 + X	22 + X	22 + Y	22 + X	22 + Y
rDNA copy number	NA	NA	123	157	132	117	79	149

*Note*: Merqury results of CQ v2.0 and CQ v3.2 were from the same pipeline used in this study. The QV and GCI data for CN1 were obtained from [10] and [33], respectively; the data for YAO were obtained from [11]. T2T, telomere-to-telomere; QV, quality value; GCI, Genome Continuity Inspector; HiFi, high-fidelity; ONT, Oxford Nanopore Technologies; NA, not available.

In addition, we evaluated CQ v3.2 using Merqury [29], a *k*-mer-based reference-free assessment pipeline, using both PCR-free pair-end and HiFi reads to identify assembly errors (Figure S4A) and switch errors between the CQ v3.2_mat and CQ v3.2_pat haplotypes (Table 1; Figure S4B). We observed a higher switch error rate in CQ v3.2 compared to CQ v2.0 when using 21-mer databases; however, with the more stringent 31-mer database, CQ v3.2 demonstrated improved performance over CQ v2.0 (Table 1**)**. This can be attributed to the fact that CQ v2.0 was assembled by phasing long reads using the read-based phasing tool WhatsHap [30] followed by separate haplotype assembly [16]. In contrast, CQ v3.2 adopted a trio binning strategy, which improves continuity while simultaneously mitigating switch errors [31]. In comparison, the switch error rate of CQ v3.2 was higher than that of T2T-YAO (Table 1). Notably, despite this slight increase, CQ v3.2’s switch error rates (0.19% and 0.20% for 21-mers; 0.22% and 0.23% for 31-mers) remained substantially lower than the average haplotype switch error rate (0.67% for 21-mers) reported among 47 high-quality diploid human genome assemblies [32]. As such, CQ v3.2 demonstrates superior completeness compared to CQ v2.0 while exhibiting significantly lower false duplication rates (Table 1, Table S4). Notably, CQ v3.2’s false duplication rate was even lower than that of T2T-YAO (Table 1).

The whole-genome QVs (*k* = 21) for CQ v3.2_mat (67.79) and CQ v3.2_pat (66.59) demonstrate significant improvement over both CQ v2.0_mat (57.42) and CQ v2.0_pat (58.15) (Figure 1B), substantially exceeding those of the first T2T diploid human genome assembly, CN1 [10] (Table 1). The 21-mer QVs of CQ v3.2 were nearly comparable to those of T2T-YAO [11] and approached the quality of T2T-CHM13 (QV = 73.94) in a parallel comparison [28]. We additionally performed Merqury QV assessments using 31-mers to compare CQ v3.2 and CQ v2.0. Due to the increased sensitivity of 31-mers, particularly in homopolymer-rich regions, the QVs (*k* = 31) were generally lower than those obtained with 21-mers (Table 1). Nevertheless, CQ v3.2 consistently maintained higher QVs than CQ v2.0 (Table 1).

Moreover, compared to CQ v2.0, the contig N50 and QV of CQ v3.2 were significantly improved (Table 1). The contig N50 values in CQ v3.2_mat and CQ v3.2_pat are 155.11 Mb and 155.17 Mb, respectively, surpassing those of CQ v2.0_mat (132.84 Mb) and CQ v2.0_pat (132.84 Mb). To further evaluate the assembly continuity of CQ v3.2, we employed two computational tools: Genome Continuity Inspector (GCI) [33] and Clipping Reveals Assembly Quality (CRAQ) [34]. Our CQ v3.2 assemblies achieved GCI scores (HiFi + ONT) of 77.76 (CQ v3.2_mat) and 76.41 (CQ v3.2_pat), demonstrating continuity comparable to T2T-CN1_pat (77.90) while showing substantial improvement over T2T-CN1_mat (66.79) (Table 1). Using CRAQ, we obtained both regional assembly quality indicators (R-AQI) and structural assembly quality indicators (S-AQI), with CQ v3.2_mat scoring R-AQI = 99.61 and S-AQI = 99.82, while CQ v3.2_pat achieved R-AQI = 99.65 and S-AQI = 99.79. According to CRAQ’s quality thresholds, an AQI score greater than 90 indicates reference-level quality [34]. We evaluated the CQ v3.2 genomes using HMM-Flagger [32], a read-mapping-based tool designed to detect mis-assemblies in diploid genomes. Based on HiFi and ONT ultralong read mappings, 99.33% and 99.17% of the CQ v3.2 assembly, respectively, were flagged as correctly assembled (Table S5). Overall, the convergence of multiple evaluation metrics provides robust evidence for the exceptional continuity and accuracy of our CQ v3.2 assemblies compared to CQ v2.0, particularly in centromeric regions (Figure 1C). A representative example is observed on chromosome 6: while the CQ v2.0_mat assembly spans 167,988,758 bp, the CQ v3.2_mat assembly extends to 173,304,238 bp. This represents a gain of 5,315,480 bp of centromeric sequence in the T2T-level assembly (Figure 1C; Table S6).

Comparative analysis of the CQ v3.2 and CQ v2.0 genomes also identified two megabase-scale inversions on chromosomes 8 and 16 (Figure 1D, Figures S5 and S6), specifically within the paternal haplotypes. These SVs were validated through GCI-based sequencing coverage plots (Figure S7) and manual verification (Figure S8), confirming their accuracy. We next used SyRI [35] to identify all inversion variants > 100 kb between CQ v3.2 and CQ v2.0. A total of 7 and 13 inversions were detected in the maternal and paternal haplotypes, respectively (Table S7), which we again confirmed with GCI and manual verification (Figures S7–S10).

The heterozygosity rate between the two CQ v3.2 haplotype genomes, calculated using the “SV count” method (https://github.com/T2T-CN1/CN1/tree/main/heterozygosity), revealed that the most diverse regions are located in the centromeres (Figure 1E). The heterozygosity rate in the centromeric regions was 5.3 times higher than that in the non-centromeric regions (on average 12.18 *vs.* 2.29 SVs per 500 kb) (Figure S11), similar to the 5.4-fold difference observed in T2T-CN1 [10]. The high heterozygosity in centromeric regions suggests that these areas are genetically diverse and may play a significant role in maintaining genetic variation within a population.

Since our genome assembly was constructed by merging data from monozygotic twins LCL5 and LCL6, we identified variants in each twin using CQ v3.2 as reference to enrich the heterozygous sites in the assembly. Our analysis detected 3663 single-nucleotide variants (SNVs) and 20,680 insertions and deletions (indels) (Table S8, available at https://github.com/BoWangXJTU/T2T-CQ/tree/main/twin_diff), including 1027 SNVs and 109 indels with allele frequencies > 0.3 located outside repeat regions (Figures S12 and S13; Table S8).

Notably, we found that the maternal haplotype contained 142 SNVs and 34 indels unique to LCL5, compared to 314 SNVs and 50 indels unique to LCL6 (Table S8). Similarly, for the paternal haplotype, 145 SNVs and 37 indels were specific to LCL5, while 426 SNVs and 68 indels were unique to LCL6 (Table S8). These comprehensive variant profiles provide a valuable resource for future studies in human family genetics [4].

### Centromeric annotations and evolutionary dynamics of novel HORs

**Figure 2:**
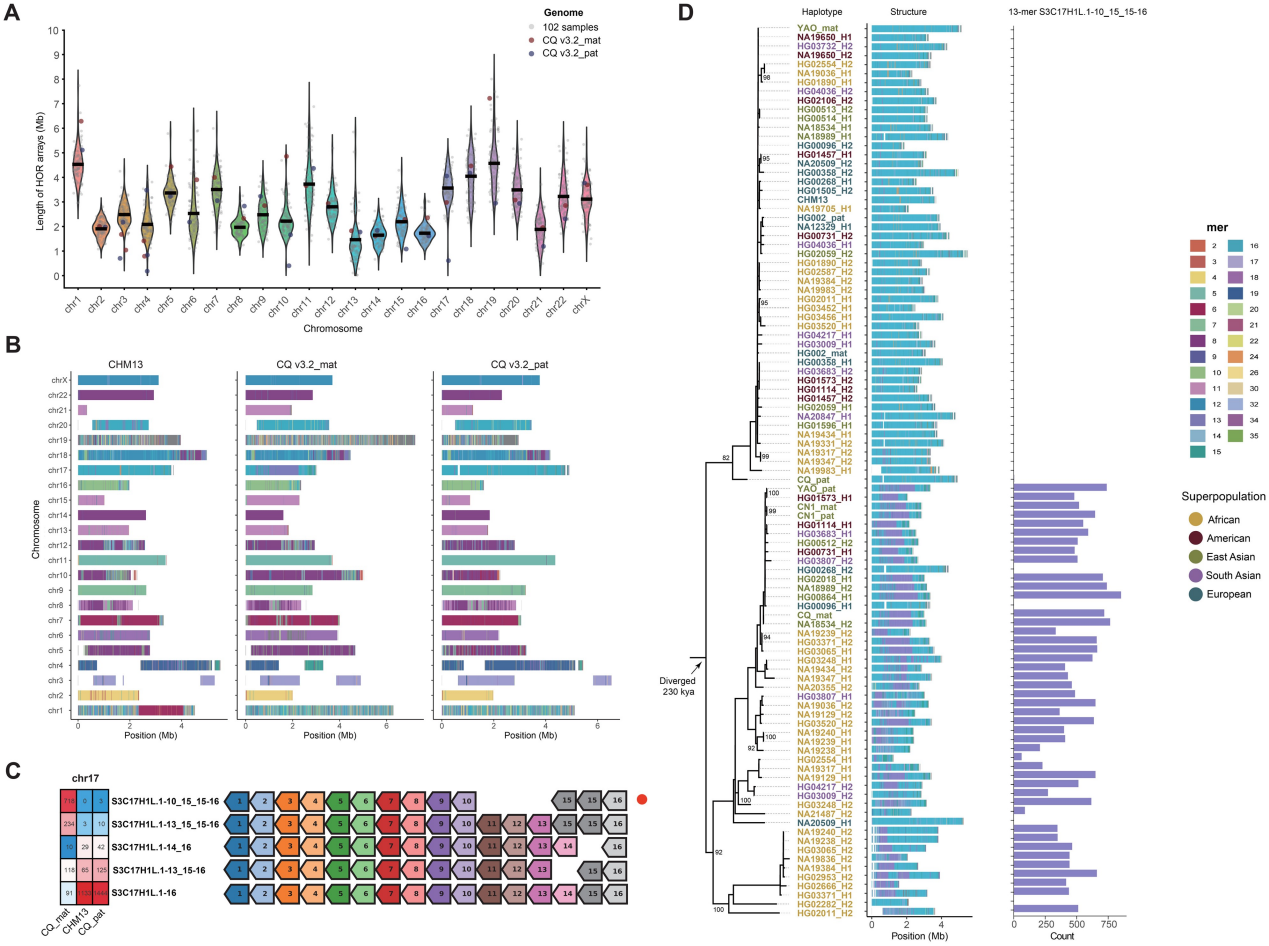
Comparative analysis of centromeric HOR length, structural organization, and evolutionary emergence of novel HORs in CQ v3.2 *vs.* diverse human genomes **A**. The length of the HOR arrays in CQ v3.2_mat (red), CQ v3.2_pat (purple), and 102 human samples (gray). The horizontal bar indicates the mean value. **B**. The HOR array structure is shown on the axes, along with the organization of each centromeric region in CHM13, CQ v3.2_mat, and CQ v3.2_pat. **C**. The heatmap showing the HOR number in chromosome 17 of CHM13, CQ v3.2_mat, and CQ v3.2_pat, along with the HOR patterns. Monomer composition is indicated by numbered arrows (color-coded by type), with the novel HOR pattern marked by a red solid circle. **D**. Maximum-likelihood phylogeny of chromosome 17 HOR arrays, with divergence time estimates supporting a monophyletic origin for novel 13-mer HORs. Nodes with ≥ 80% bootstrap support are labeled numerically. Bar plots quantify 13-mer HOR abundance across five superpopulations (African, American, East Asian, South Asian, European), with haplotype colors corresponding to their geographic origin. HOR, higher-order repeat; kya, thousand years ago.

With the generation of T2T-CQ, we were able to characterize the landscape of centromeric sequences in the Chinese Quartet. First, we annotated the centromeric α-satellite regions in CQ v3.2_mat and CQ v3.2_pat using the CenMAP pipeline, which ranged from 2.7 Mb to 10.2 Mb in CQ v3.2_mat (Table S9) and from 2.5 Mb to 8.4 Mb in CQ v3.2_pat (Table S10). Sequence identity maps generated using StainedGlass [36] are displayed for each centromere in both CQ v3.2_mat and CQ v3.2_pat (Figures S14–S17). The length of the α-satellite HOR array is well known to exhibit variation across human populations [3,37]. Accordingly, we annotated the HOR array lengths in both haplotypes and compared them with the HOR lengths of 102 additional human samples [37] (detailed in Materials and methods) (**Figure 2**A). We found that the HOR length of CQ v3.2_mat ranged from 787 kb to 6.3 Mb (Table S11), while that of CQ v3.2_pat ranged from 183 kb to 5.1 Mb (Table S12). Interestingly, the HOR lengths of the two haplotypes of CQ differed significantly, with the exception of chromosome 2, where the HOR lengths were similar (1,999,710 bp in CQ v3.2_mat and 1,978,117 bp in CQ v3.2_pat), both close to the mean value (Figure 2A).

We subsequently visualized and compared the α-satellite HOR array structures between the CQ v3.2 haplotypes (Chinese) and T2T-CHM13 (European) (Figure 2B), finding substantial differences in HOR composition between them. To assess population-level variation, we systematically analyzed divergence patterns across centromeric contigs, including both European–Chinese comparisons and intra-Chinese individual variations (see Materials and methods). Notably, no significant correlation was detected between intra- and inter-population variation patterns (Figures S18 and S19), which may be explained by the dynamic evolutionary behavior of HORs [3].

For chromosome 1, the T2T-CHM13 assembly exhibits nearly 2 Mb of unique HOR structures compared to both CQ v3.2_mat and CQ v3.2_pat (Figure 2B). These regions predominantly comprise 2-mer HORs (S1C1/5/19H1L.5_6/4 and S1C1/5/19H1L.5-6) and a 6-mer HOR (S1C1/5/19H1L.1-5_6/4). Strikingly, the S1C1/5/19H1L.5_6/4 motif appears to be entirely absent from chromosome 1 in both haplotypes of CQ v3.2, CN1, and HG002 as well as YAO_pat (Table S13). Interestingly, YAO_mat shows a HOR structure remarkably similar to T2T-CHM13 (Figure S20), suggesting that these two haplotypes may have independently acquired and expanded hybrid monomers. This pattern parallels the observed hybridization between S1C1/5/19H1L monomer 6 and monomer 4.

For chromosome 17, a unique region of less than 2 Mb is observed in CQ v3.2_mat, composed of the 13-mer HOR S3C17H1L.1-10_15_15-16 (Figure 2B). The canonical HOR pattern for chromosome 17 is S3C17H1L.1-16 [10], whereas S3C17H1L.1-10_15_15-16 lacks monomers 11, 12, 13, and 14, and has monomer 15 duplicated once (Figure 2C). The S3C17H1L.1-10_15_15-16 motif appears exclusively in CQ v3.2_mat, while it is entirely absent in T2T-CHM13 and present in only three copies in CQ v3.2_pat (Figure 2C). This suggests that S3C17H1L.1-10_15_15-16 represents a novel HOR specific to CQ v3.2 (Figure 2B and C). Interestingly, both alleles of chromosome 17 in T2T-CN1 and one haplotype in T2T-YAO were found to contain this 13-mer HOR [10] (Figure S21), initially suggesting a potential association with the Han Chinese population.

To test this hypothesis, we analyzed 64 additional genomes from HGSVC3, HG002, and T2T-YAO, representing diverse superpopulations (African, American, European, South Asian, and other East Asian groups). Among 99 fully assembled chromosome 17 centromeres subjected to HOR annotation, 43 haplotypes (43/99) carried the S3C17H1L.1-10_15_15-16 motif across all superpopulations (Figure 2D**)**, indicating that this “novel 13-mer HOR” is not unique to Han Chinese. To investigate the evolutionary dynamics underlying these HOR arrays — particularly the emergence of novel HORs — we constructed phylogenetic trees using chimpanzee sequences as an outgroup and estimated divergence times, assuming a human-chimpanzee split at 6 million years ago (Figure 2D). Phylogenetic analysis revealed two distinct clusters, with haplotypes containing the novel HORs predominantly forming a monophyletic clade (Figure 2D). We estimate that these novel HORs of chromosome 17 arose approximately 230 thousand years ago (kya). While similar HOR evolutionary patterns were reported for chromosomes 5, 10, 11, and 12 [3], our findings on chromosome 17 uncover a more widespread distribution of novel HORs across diverse populations, suggesting additional layers of centromeric evolution.

### Twin-based validation of centromeric assembly fidelity

Our CQ material differs from the CN1 and YAO assemblies in comprising sequencing data from two monozygotic twins, representing two distinct trios at the data level. This makes them particularly suitable for evaluating trio binning assembly software, especially in complex centromeric regions. In our systematic evaluation using ONT ultralong coverages (ranging from 5× to a maximum coverage of 40.4× for LCL5 and 27.2× for LCL6, with HiFi reads normalized to 50× for both samples), we employed two state-of-the-art assemblers (hifiasm v0.25.0 and Verkko v2.2.1) to assess centromeric region assembly robustness. The results demonstrated that both assemblers generally produced improved contig N50 values with increasing ONT ultralong coverage, enabling more complete centromere assembly in both twins. Notably, while LCL5 assemblies in hifiasm showed a slight N50 decrease beyond 20× ONT coverage (at 25× and 40.4×), they still yielded more complete centromeres than at 20× (Table S14).

We next performed comparative analyses of complete centromere lengths across different coverage depths (Figure S22A and B), revealing both assemblers to be comparably robust. Further detailed analysis of maximum length variations for each centromere at ≥ 15× sequencing depth showed that hifiasm assemblies exhibited a maximum length difference of 16,629 bp in LCL5 (chromosome 10 paternal haplotype) and 182,966 bp in LCL6 (chromosome 15 paternal haplotype) (Table S15). Similarly, Verkko assemblies displayed maximum differences of 28,880 bp in LCL5 (chromosome 7 maternal haplotype) and 75,499 bp in LCL6 (chromosome 11 paternal haplotype).

These coverage-dependent variations were significantly smaller than allelic differences. For instance, in LCL6’s chromosome 15, while maternal/paternal haplotype differences reached 1.3 Mb, the maximum length variations within maternal (37,284 bp) and paternal (8019 bp) haplotypes were orders of magnitude smaller.

Our findings demonstrate that both assemblers can control centromeric assembly errors within 300 kb (often to just a few base pairs), minimizing impact on sample haplotype comparisons. However, for base-level or HOR-level analyses, further improvements in assembly precision remain necessary.

## Conclusion

In summary, this study significantly advances genomic research by presenting a high-quality T2T diploid assembly of the Chinese Quartet. Leveraging cutting-edge sequencing technologies, we identified novel HORs in centromeric regions and elucidated their dynamic evolutionary patterns, including expansions and contractions. Additionally, we systematically evaluated the robustness of state-of-the-art assembly tools in centromere reconstruction using twin sequencing data. These findings deepen our understanding of centromere structural diversity and evolution. Moreover, this work establishes a critical genomic resource for constructing a comprehensive T2T pan-Chinese reference genome, which will facilitate population-scale genomics research and precision medicine initiatives [38,39].

## Materials and methods

### Sample collection

The Chinese Quartet family, comprising a 60-year-old father (LCL7), 60-year-old mother (LCL8), and their two 30-year-old monozygotic twin daughters (LCL5 and LCL6), all from the Fudan Taizhou cohort, are Certified Reference Materials according to the State Administration for Market Regulation in China (reference Nos. GBW09900–GBW09903). The process of establishing the cell lines was described in a prior study [40].

### ONT ultralong library preparation and sequencing

ONT ultralong DNA was extracted using the GrandOmics Genomic DNA Kit (Catalog No. SQK-LSK114, GrandOmics, Wuhan, China) following the manufacturer’s guidelines. The quality and quantity of the total DNA were assessed to ensure suitability for ultralong Nanopore library construction, which was performed according to the established protocols [41]. DNA libraries (approximately 400 ng) were constructed and sequenced on the PromethION platform (Oxford Nanopore Technologies) at the Genome Center of GrandOmics (Wuhan, China).

### Hi-C sequencing

The Hi-C library was prepared from cross-linked chromatins of the cells following a standard Hi-C protocol [42]. The library was subsequently sequenced on the MGI-2000 platform. The Hi-C sequencing data were used to map the assembled genome using Juicer (v1.6) [43]. The assemblies were then manually reviewed and visualized using Juicebox (v1.11.08) [44].

### Genome assembly

Since monozygotic twins are generally considered genetically identical, with only limited somatic substitutions [45], we merged the sequencing reads from the two children to generate a high-quality haplotype-resolved genome [16]. The initial assemblies were constructed using state-of-the-art tools, Verkko (v2.2.1) [22,23] and hifiasm (v0.19.9) [24], in trio mode. The scaffolding of the Verkko assemblies was enhanced using the hifiasm assembly, as the contig N50 of the Verkko-assembled genome (131.8 Mb) was greater than that of hifiasm (107.5 Mb). For each haplotype, hifiasm and Verkko assemblies were assigned to their respective chromosomes and aligned to the CHM13 reference genome (Figure S1A). Within each chromosome, contigs were merged into longer scaffolds based on overlaps between hifiasm and Verkko assemblies. Additionally, supplementary assemblies were generated using hifiasm (v0.24.0) with the “--ONT” parameter, incorporating canu-based [46] binned ONT reads. The gaps were initially filled using TGS-GapCloser (v1.2.1) [25], leveraging the hifiasm assemblies with ONT data and binned ONT reads. The highly repetitive nature of rDNA, combined with heterozygosity, presents significant challenges for resolution [1]. Therefore, only the rDNA CN was estimated to populate each chromosome with corresponding identical copies [10]. The mitochondrial genome was assembled using Unicycler (v0.5.1) [47].

### Genome polishing

Since our genome assembly was performed using the trio binning approach, the two haplotype assembly results were polished separately. First, yak (v0.1-r56; https://github.com/lh3/yak) was used to bin HiFi reads based on parental information. Subsequently, the “getseq” function from fxTools (v0.1.0; https://github.com/moold/fxTools) was employed to extract the binned HiFi reads. The “Racon + Merfin” pipeline was then executed to generate the HiFi mapping file [28], “racon.meryl.iter_1.winnowmap.bam”. Finally, the NextPolish2 workflow was run to produce the polished genome files [27]. To identify and correct potential SV errors, we implemented the “Find SV-like errors” step from the T2T polishing pipeline (https://github.com/arangrhie/T2T-Polish) to refine our genome assembly [28].

### Genome assessment

To construct a hybrid *k*-mer database, we employed Meryl (v1.4.1) to extract 21-mers and 31-mers from both Illumina PCR-free paired-end data and HiFi reads obtained from the twins (Table S1). Due to the excessively high Illumina sequencing depth (> 340×) in the twins’ data that could generate numerous sequencing errors, we downsampled the Illumina dataset to approximately 100×. The database was generated using the following sequence of commands:

meryl greater-than 1 twins.hifi.meryl output twins.hifi.gt1.meryl

meryl greater-than 1 twins.k31mer.meryl output twins.k31mer.gt1.meryl

meryl divide-round 3 twins.k31mer.gt1.meryl output twins.k31mer.gt1.divideRound3.meryl

meryl union-max twins.hifi.gt1.meryl twins.k31mer.gt1.divideRound3.meryl output hybrid.meryl

Parental datasets, consisting of Illumina PCR-free paired-end sequences, were obtained from LCL7 (father) and LCL8 (mother) (Table S1**)**. Subsequently, Merqury was employed to evaluate the assemblies [29], following the methodology outlined in previous studies [10,11]. In addition to *k*-mer-based metrics, assembly continuity was assessed using GCI (v1.0) [33] and CRAQ (v1.0.9) [34], two read-mapping-based tools that incorporate both HiFi and ONT ultralong reads. We subsequently employed the HMM-Flagger (v1.1.0) tool to identify erroneous regions, false duplications, and collapsed segments in the diploid genome assembly [48], while also detecting structurally correct haploid regions. HMM-Flagger analysis was performed separately using read depth information, HiFi reads, and ONT ultralong reads.

### Genomic variation

To comprehensively identify the full spectrum of heterozygosity between the CHM13 and the diploid T2T-CQ genomes, we performed a direct comparison of these three assemblies using minimap2 (v2.20-r1061) [49] with the following parameters: -x asm5 -t 64 --cs. The paf2delta.pl script (https://github.com/gorliver/paf2delta) was used to convert the PAF format into the delta format. Subsequently, the “show-snps” function from MUMmer (v4.0.0rc1) [50] and the “Transform-SNP” function from GenomeSyn [51] were employed to generate SNP results. Additionally, the “show-diff” function from MUMmer and the “Transform-PAV” function from GenomeSyn were utilized to produce Presence-Absence Variation (PAV) results. Following this, GenomeSyn was utilized to conduct a whole-genome comparison between the CHM13 genome and the assembled genome in this study. SVs > 100 kb, specifically inversions, between the CQ v3.2 and CQ v2.0 genome assemblies were identified using SyRI (v1.7.0) [35]. For heterozygous site comparison in twins, phased HiFi reads from both twins were aligned to the maternal and paternal haplotypes of the CQ v3.2 genome using “minimap2 + VarScan v2.4.6” variant calling pipeline [49,52].

### Centromere analysis

CenMAP (v0.2.4; https://github.com/logsdon-lab/CenMAP), a centromere mapping and annotation pipeline designed for T2T human genome assemblies, was utilized to predict potential centromeric regions in the genome. The α-satellite sequences were annotated using the pipeline hmmer-run.sh (https://github.com/fedorrik/HumAS-HMMER_for_AnVIL) with the hmm file “AS-HORs-hmmer3.3.2-120124.hmm”. Subsequently, HOR SVs were identified using the tool StV (https://github.com/fedorrik/stv). StainedGlass (v0.6) was employed to visualize the centromeric regions [36], and custom R scripts were utilized to plot the HOR length and StV annotations. The HOR length was calculated using “censtats” from CenMAP (v0.2.4). The HOR lengths of 102 additional human samples — including 22 African individuals from Human Pangenome Reference Consortium (HPRC) [32], 16 Latin American individuals (HPRC), 62 East Asian individuals (4 from HPRC and 58 from Chinese Pangenome Consortium [48]), and 1 South Asian individual (HPRC), plus CHM13 — were obtained from our previous study [37]. For comprehensive analysis of centromere structure and phylogeny, we integrated multiple recently available genomic resources, including 64 Human Genome Structural Variation Consortium Phase 3 (HGSVC3) genomes [12] (excluding HG002, for which we used v1.1 from https://github.com/marbl/HG002), YAO v1.1 [11], CN1 v1.0.1 [10], CHM13 [1], and CQ v3.2. In total, this dataset comprised 69 samples representing 137 haplotypes.

### Phylogenetic analysis

To investigate the phylogenetic relationships and divergence times of centromeric regions from chromosome 17 across 69 diverse human genomes (encompassing 137 haplotypes), we conducted phylogenetic analyses following a modified approach from [3]. We first identified contigs containing fully assembled centromeres using CenMAP (v0.2.4), then aligned each to the unique q-arm reference region (chr17:28,103,337–28,138,133; CHM13 v2.0) using unimap (https://github.com/lh3/unimap). For the outgroup comparison, we included chimpanzee sequences [53] aligned to the same unique region. Multiple sequence alignment was performed using MAFFT (v7.526) [54] (with parameter “--auto”), followed by maximum-likelihood phylogenetic reconstruction with IQ-TREE (v2.0.3) [55] (with parameter “-m MFP -B 1000 --alrt 1000 -T AUTO”). The resulting trees were visualized in FigTree (v1.4.4; http://tree.bio.ed.ac.uk/software/figtree/). Divergence time estimation was conducted using r8s (v1.81) [56] with the penalized likelihood method and the truncated Newton algorithm, applying a molecular clock calibrated to a human-chimpanzee divergence time of 6 million years [3].

### Sequence identity analysis across centromeric regions

To assess sequence conservation and population divergence, we compared centromeric regions between European and Chinese haplotypes. For the Chinese population, we analyzed two haplotypes each from the CN1 v1.0.1 [10], YAO v1.1 [11], and CQ v3.2 assemblies, while for the European population, we examined CHM13 v2.0 along with two haplotypes each from the HG002 v1.1, HG00171 (HGSVC3), and HG00268 (HGSVC3) assemblies. The European and Chinese references were CHM13 v2.0 and YAO v1.1_mat, respectively. Pairwise alignment of centromeric contigs was performed using minimap2 with optimized parameters (-I 15G -K 8G -ax asm20 --secondary = no --eqx -s 2500) [3], retaining only primary alignments (SAMtools flag 4). We then partitioned alignments into consecutive 10-kb non-overlapping windows relative to the reference genomes and calculated sequence identity per window as: (matches)/(matches + mismatches + insertions + deletions). This approach allowed systematic comparison of intra- and inter-population variation while accounting for structural differences in centromeric regions [3].

### Evaluation of assembly fidelity in centromeric regions

To evaluate the robustness of centromere assembly in two state-of-the-art genome assemblers [hifiasm (v0.25.0) and Verkko (v2.2.1)], we employed 50× HiFi reads as the baseline and tested with ONT ultralong reads (> 100 kb) at varying coverages (5×, 10×, 15×, 20×, 25×, and maximum coverage). Random subsampling of sequencing reads was performed using Rasusa [57] with the following parameters: reads --coverage specific coverage --genome-size 3Gb. Both assemblers were run in trio binning mode using default parameters and were tested on twin samples LCL5 and LCL6. For centromere assessment, we identified complete centromeres using CenMAP (v0.2.4) and compared their lengths to determine the robustness of each assembly approach.

### Estimation of rDNA CN using ddPCR

The ddPCR experimental protocol followed a previously published method [10], with the modification that the single-copy gene *RPPH1* was used instead of the *TBP1* gene. The rDNA forward primer was 5′-AACGTGAGCTGGGTTTAG-3′, the rDNA reverse primer was 5′-CTCGTACTGAGCAGGATTAC-3′, and the rDNA probe was 5′-CACATCATCAGTAGGGT-3′. The forward primer, reverse primer, and probe for *RPPH1* were 5′-GAGGGAAGCTCATCAGTGG-3′, 5′-CCCTAGTCTCAGACCTTCC-3′, and 5′-CCACGAGCTGAGTGC-3′, respectively. The rDNA CN was calculated as the ratio of rDNA copies/µl to *RPPH1* copies/µl.

### rDNA analysis

Ribotin was used to extract binned ONT reads containing rDNA sequences [26]. Subsequently, the extracted ONT reads were mapped to the major morphs of CHM13 (https://github.com/maickrau/ribotin_paper_experiments) using minimap2 (v2.20-r1061) [49]. We calculated the ONT read counts and base counts for each major morph of CHM13, determined the rDNA CN for each morph, and used this information to fill the rDNA regions in the genome assembly (https://github.com/BoWangXJTU/CQ_T2T/tree/main/04.rDNA_analysis).

## Ethical statement

The Quartet Project received approval from the Institutional Review Board of the School of Life Sciences at Fudan University, China (Approval No. BE2050). The study was conducted in accordance with the principles outlined in the Declaration of Helsinki. Four healthy volunteers from a family quartet, participating in the Taizhou Longitudinal Study in Taizhou, Jiangsu Province, China, were enrolled. Peripheral blood samples were collected from these individuals to establish human immortalized B-lymphoblastoid cell lines. All four donors provided signed informed consent forms prior to participation.

## Code availability

The pipelines and code for genome assembly, polishing, centromere analysis, and rDNA analysis are available on GitHub (https://github.com/BoWangXJTU/T2T-CQ). The code has also been submitted to BioCode at the National Genomics Data Center (NGDC), China National Center for Bioinformation (CNCB) (BioCode: BT007908), which is publicly accessible at https://ngdc.cncb.ac.cn/biocode/tools/BT007908.

## Supplementary Material

qzaf118_Supplementary_Data

## Data Availability

The raw sequencing data of the Chinese Quartet have been deposited in the Genome Sequence Archive for Human [58] at the NGDC, CNCB (GSA-Human: HRA010594), and are publicly accessible at https://ngdc.cncb.ac.cn/gsa-human. The CQ v3.2 genome sequences have been deposited in the Genome Warehouse [59] at the NGDC, CNCB (GWH: GWHFQEY00000000.1 and GWHFQEX00000000.1), and are publicly accessible at https://ngdc.cncb.ac.cn/gwh.

## CRediT author statement

**Bo Wang:** Investigation, Writing – original draft, Methodology, Visualization, Funding acquisition. **Peng Jia:** Investigation, Methodology, Visualization, Writing – review & editing. **Stephen J. Bush:** Writing – review & editing. **Xia Wang:** Validation, Investigation. **Yi Yang:** Validation, Investigation. **Yu Zhang:** Validation, Investigation. **Shijie Wan:** Methodology, Visualization. **Xiaofei Yang:** Methodology, Funding acquisition. **Pengyu Zhang:** Methodology. **Yuanting Zheng:** Investigation, Data curation. **Leming Shi:** Investigation, Data curation. **Lianhua Dong:** Conceptualization, Supervision, Investigation, Data curation, Writing – review & editing, Resources, Funding acquisition. **Kai Ye:** Conceptualization, Supervision, Methodology, Writing – review & editing, Resources, Funding acquisition. All authors have read and approved the final manuscript.
