# Supplementary material for: A Telomere-to-telomere Diploid Reference Genome and Centromere Structure of the Chinese Quartet
Source: Genomics Proteomics Bioinformatics. 2025 Nov 26;23(6):qzaf118. doi: 10.1093/gpbjnl/qzaf118 (PMC13075991; doi:10.1093/gpbjnl/qzaf118)
Supplement: qzaf118_Supplementary_Data [file qzaf118_supplementary_data.zip › Supplementary material captions.docx]

**Figure S1 Illustration of the process of merging hifiasm and Verkko assemblies, followed by subsequent gap-filling steps**

**A.** The hifiasm and Verkko assemblies for maternal chromosome 11 were aligned to CHM13 (middle) to organize and orient the contigs. The red block in CHM13 indicates the centromeric region. **B.** The gap in the maternal chromosome 1 was closed using hifiasm assemblies in ONT mode with binned ONT ultralong reads.

**Figure S2**  **Comparative IGV screenshots demonstrating structural variant refinement through polishing**

IGV, Integrative Genomics Viewer.

**Figure S3**  **The Hi-C contact maps of the CQ v3.2 maternal (A) and paternal (B) haplotypes**

Hi-C, high-throughput chromosome conformation capture.

**Figure S4 The quality of the diploid CQ v3.2 assembly**

**A.** The spectrum plots of 31-mers in the assembly and reads for evaluating assembly errors. **B.** Hap-mer blob plot of the CQ v3.2 assembly. mat, maternal haplotype. pat, paternal haplotype.

**Figure S5 Karyotype alignments of CQ v3.2 maternal (top) and CQ v2.0 maternal (bottom) haplotypes**

Blue regions represent centromeric regions.

**Figure S6 Karyotype alignments of CQ v3.2 paternal (top) and CQ v2.0 paternal (bottom) haplotypes**

Blue regions represent centromeric regions.

**Figure S7 GCI coverage plots validate inversions in CQ v3.2 assemblies differing from CQ v2.0**

GCI, genome continuity inspector.

**Figure S8 IGV map of binned ONT reads mapped to CQ v3.2 and CQ v2.0**

We manually confirmed that the large inversions between chr8 and chr16 of the paternal haplotypes in CQ v3.2 and CQ v2.0 were due to assembly errors in CQ v2.0, with CQ v3.2 being correct.

**Figure S9 Four IGV maps of binned ONT reads aligned to the CQ v3.2 and CQ v2.0 maternal haplotypes, which demonstrate the correction of assembly errors in CQ v2.0, specifically for inversions greater than 100 kb**

**Figure S10 Four IGV maps of binned ONT reads aligned to the CQ v3.2 and CQ v2.0 paternal haplotypes, which demonstrate the correction of assembly errors in CQ v2.0, specifically for inversions greater than 100 kb**

**Figure S11 Heterozygosity comparison between centromeric and non-centromeric regions**

The heterozygosity rate was calculated as the SV count in each 500 kb window. Red squares represent the mean values of the data. This comparison highlights the significantly higher heterozygosity in centromeric regions compared to non-centromeric regions.

**Figure S12 Comparison of heterozygous SNV and indel allele frequencies in twins**

SNV, single nucleotide variants; indel, insertions/deletions.

**Figure S13 Comparison of heterozygous SNV and indel allele frequencies in twins**

**Figure S14 StainedGlass plots of the CEN regions of chromosomes 1 to 6 in CQ v3.2**

CEN, centromeric

**Figure S15 StainedGlass plots of the CEN regions of chromosomes 7 to 12 in CQ v3.2**

**Figure S16 StainedGlass plots of the CEN regions of chromosomes 13 to 18 in CQ v3.2**

**Figure S17 StainedGlass plots of the CEN regions of chromosomes 19 to 22 and chromosome X in CQ v3.2**

**Figure S18** **Scatter plots of centromere sequence identity between CHM13 (y-axis) and YAO_mat (x-axis) reference genomes compared to CHN and EUR population samples**

CHN, Chinese; EUR, European.

**Figure S19 Centromere sequence identity patterns between CHN and EUR populations, including within-population comparisons**

**Figure S20 Higher-order repeat array structures of the chromosome 1 centromeric region, compared among the CHM13, HG002, CN1, CQ, and YAO genomes**

**Figure S21**  **Higher-order repeat array structures of the chromosome 17 centromeric region, compared among the CHM13, HG002, CN1, CQ, and YAO genomes**

**Figure S22**  **Comparative analysis of complete centromere lengths at varying coverage depths in LCL5 and LCL6 using both hifiasm (A) and verkko (B) assemblers**

**Table S1 Sequencing depth statistics used in this study**

**Table S2 Predicted rDNA copy numbers for the five short arms of acrocentric chromosomes in the CQ v3.2 maternal haplotype**

**Table S3 Predicted rDNA copy numbers for the five short arms of acrocentric chromosomes in the CQ v3.2 paternal haplotype**

**Table S4 Completeness statistics of CQ v3.2 genome assemblies**

**Table S5**  **Performance assessment of HMM-flagger for CQ v3.2 diploid genome assemblies**

**Table S6**  **Comparative chromosome lengths of CQ v3.2 and CQ v2.0 genome assemblies**

**Table S7 Coordinates of inversion variants (> 100 kb) identified between the CQ v2.0 and CQ v3.2 genomes**

**Table S8**  **Heterozygous sites comparison in the twin cell lines LCL5 and LCL6**

**Table S9 CQ v3.2 maternal haplotype centromeric regions and their lengths**

**Table S10 CQ v3.2 paternal haplotype centromeric regions and their lengths**

**Table S11 CQ v3.2 maternal haplotype centromeric higher-order repeat coordinates**

**Table S12 CQ v3.2 paternal haplotype centromeric higher-order repeat coordinates**

**Table S13 Higher-order repeat copies of the chromosome 1 centromeric region, compared among the CHM13, HG002, CN1, CQ, and YAO genomes**

**Table S14 Contig N50 and complete centromere statistics in the LCL5/LCL6 genomes assembled with hifiasm and verkko at different ONT coverage levels (> 100 kb)**

**Table S15 Complete centromere length distributions (bp) and maximum size variation in the LCL5/LCL6 genomes assembled with hifiasm and verkko at different ONT (> 100 kb) coverage depths**
