# Supplementary figures and images for: A Telomere-to-telomere Diploid Reference Genome and Centromere Structure of the Chinese Quartet
Source: Genomics Proteomics Bioinformatics. 2025 Nov 26;23(6):qzaf118. doi: 10.1093/gpbjnl/qzaf118 (PMC13075991; doi:10.1093/gpbjnl/qzaf118)

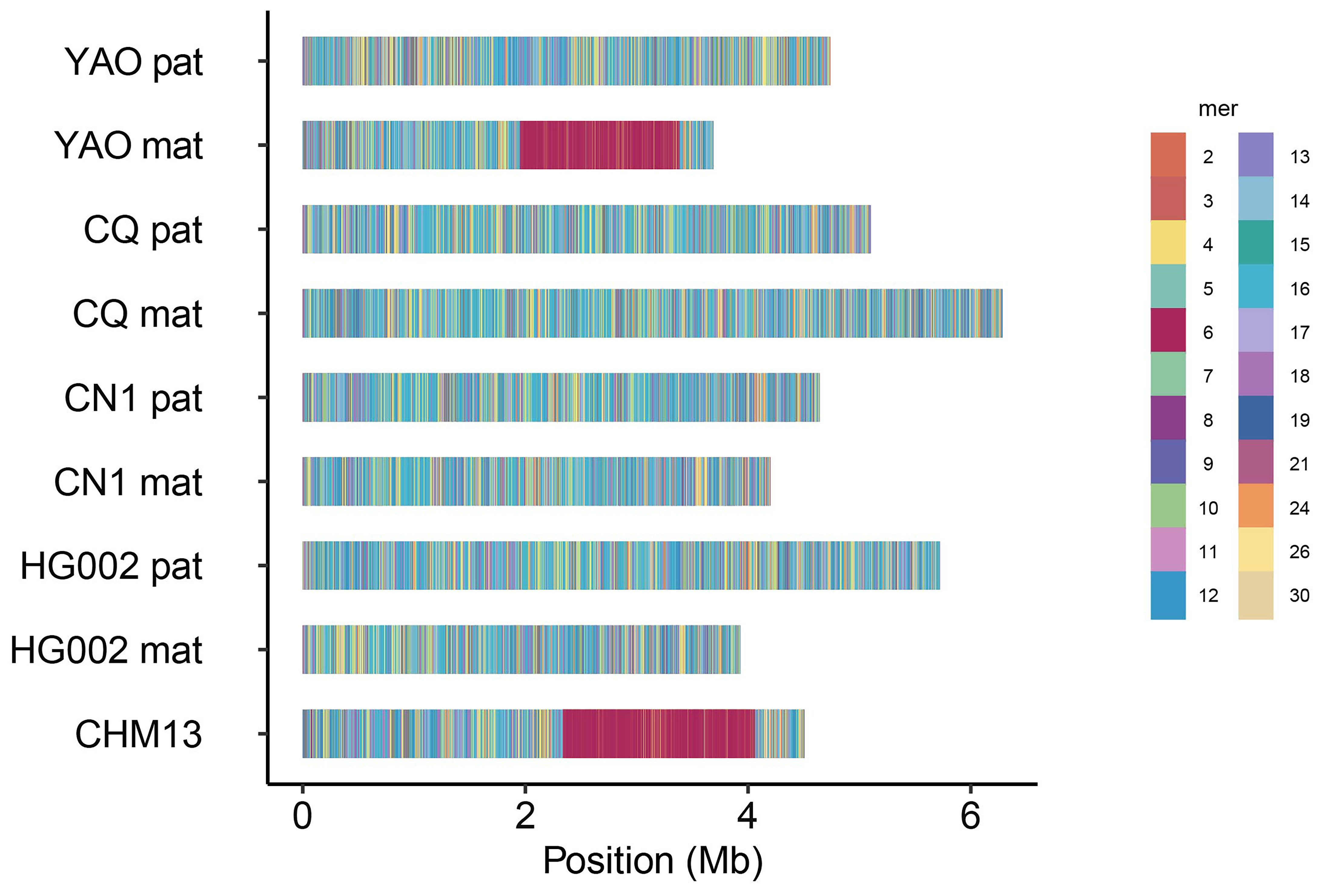

Supplement: qzaf118_Supplementary_Data [file qzaf118_supplementary_data.zip › Figure S20.jpg]

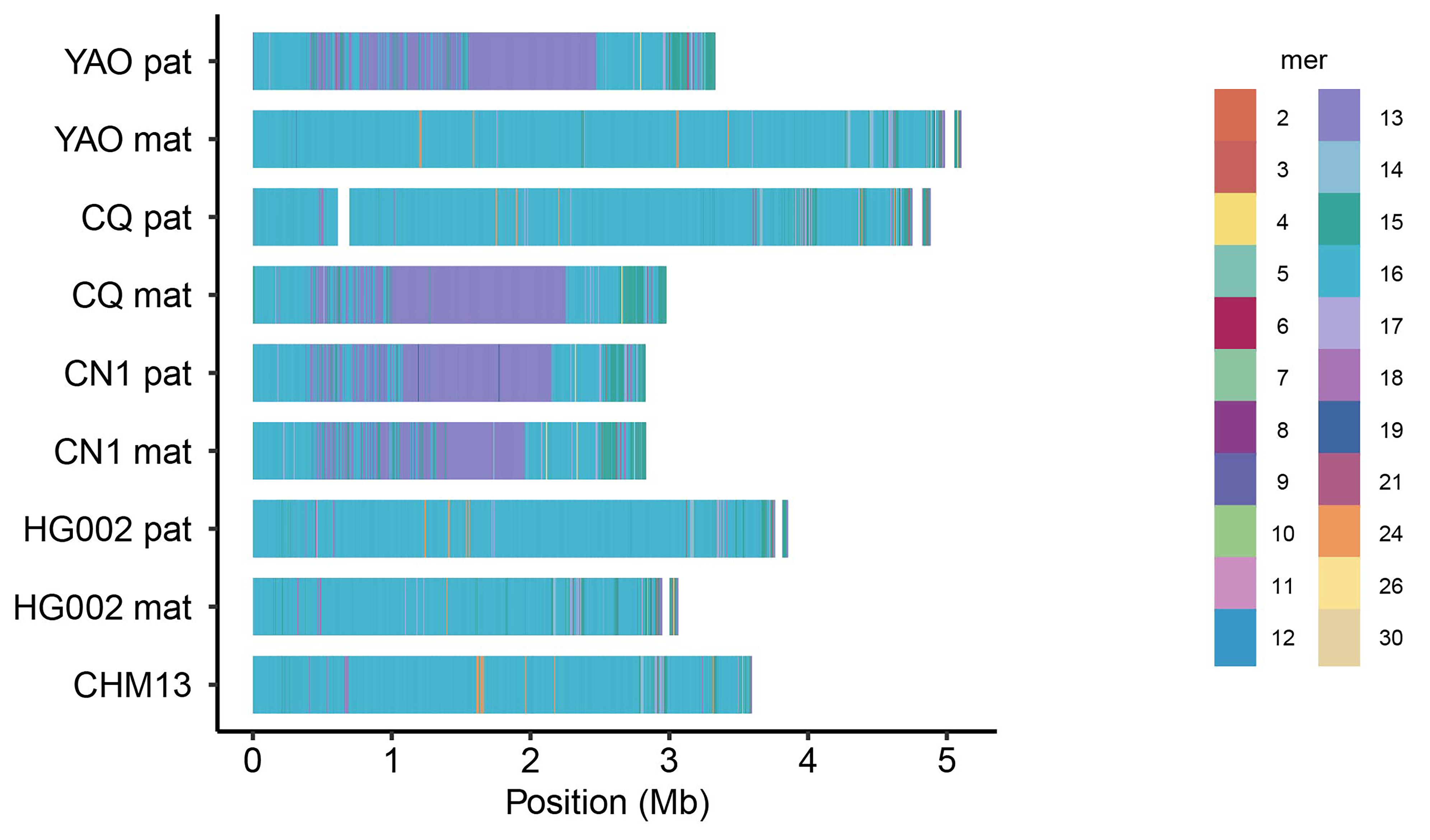

Supplement: qzaf118_Supplementary_Data [file qzaf118_supplementary_data.zip › Figure S21.jpg]

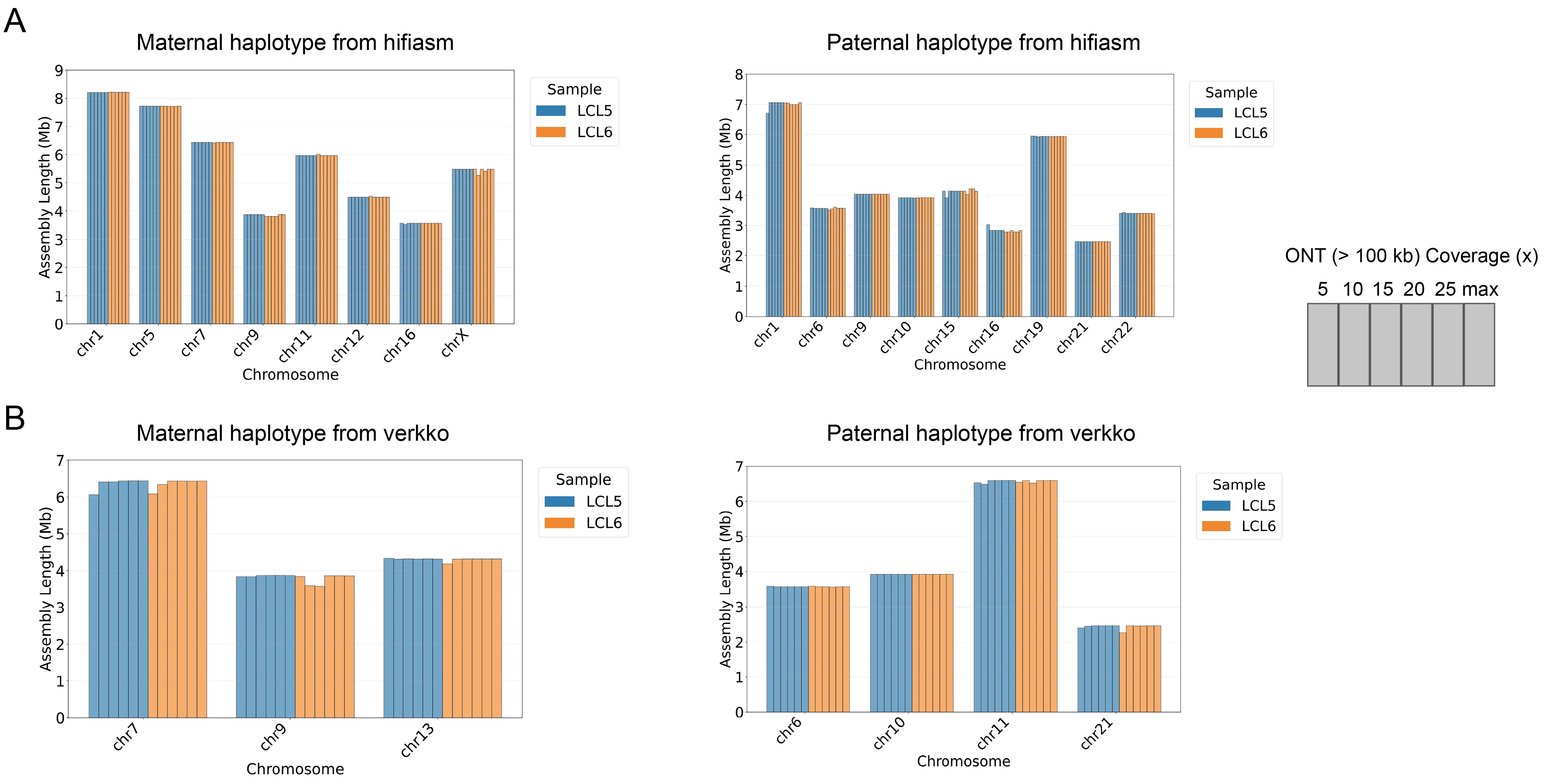

Supplement: qzaf118_Supplementary_Data [file qzaf118_supplementary_data.zip › Figure S22.jpg]

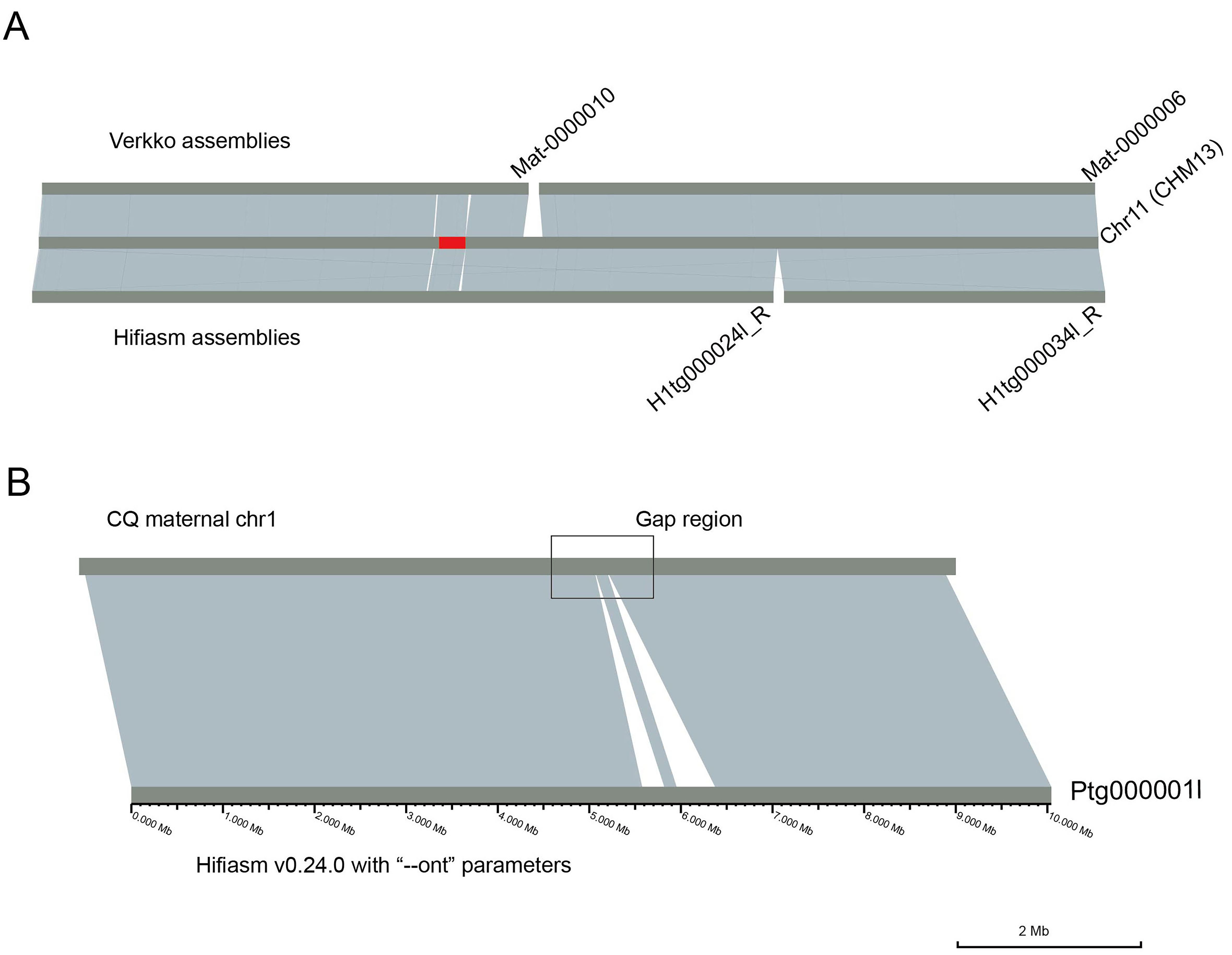

Supplement: qzaf118_Supplementary_Data [file qzaf118_supplementary_data.zip › Figure S1.jpg]

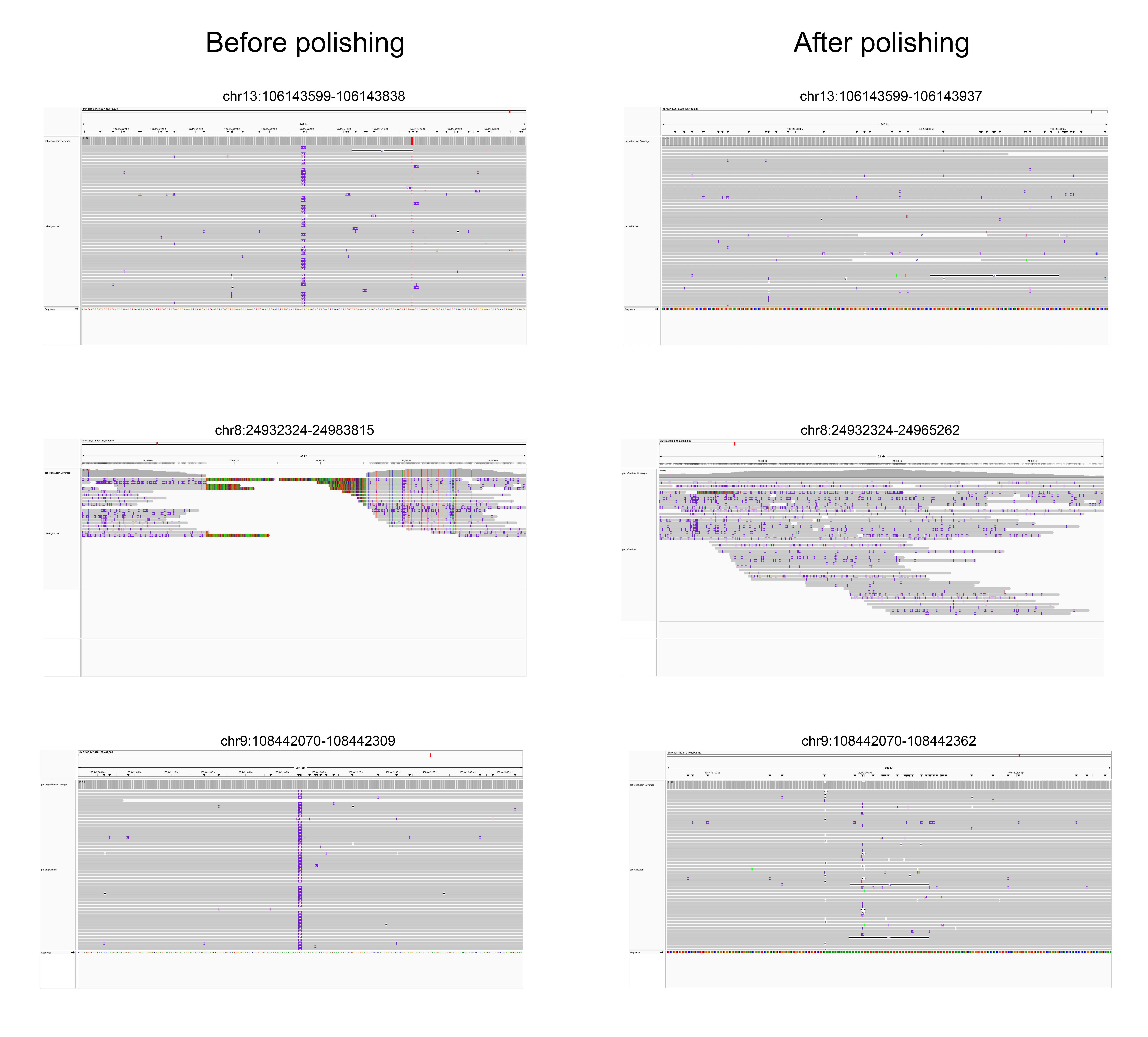

Supplement: qzaf118_Supplementary_Data [file qzaf118_supplementary_data.zip › Figure S2.jpg]

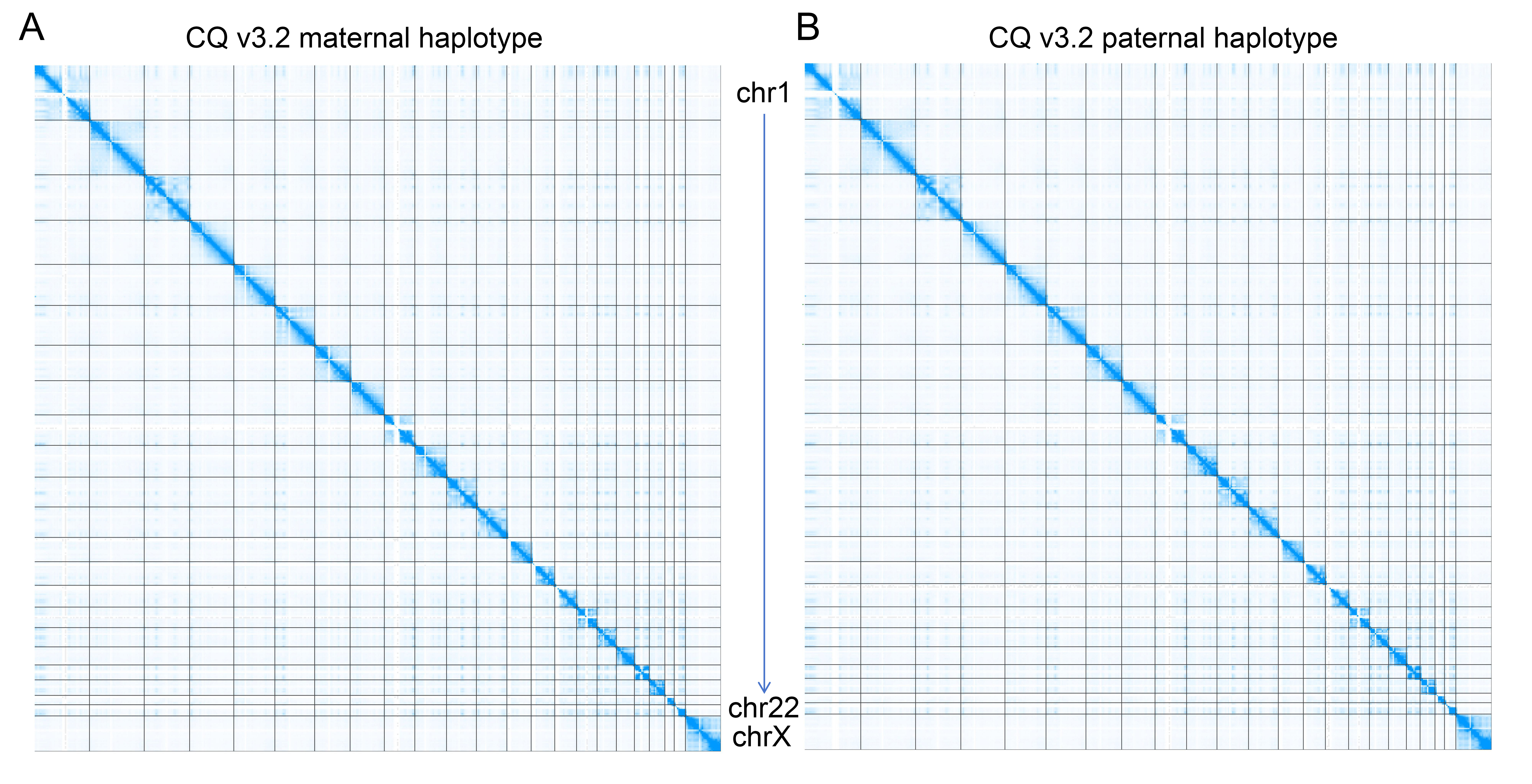

Supplement: qzaf118_Supplementary_Data [file qzaf118_supplementary_data.zip › Figure S3.jpg]

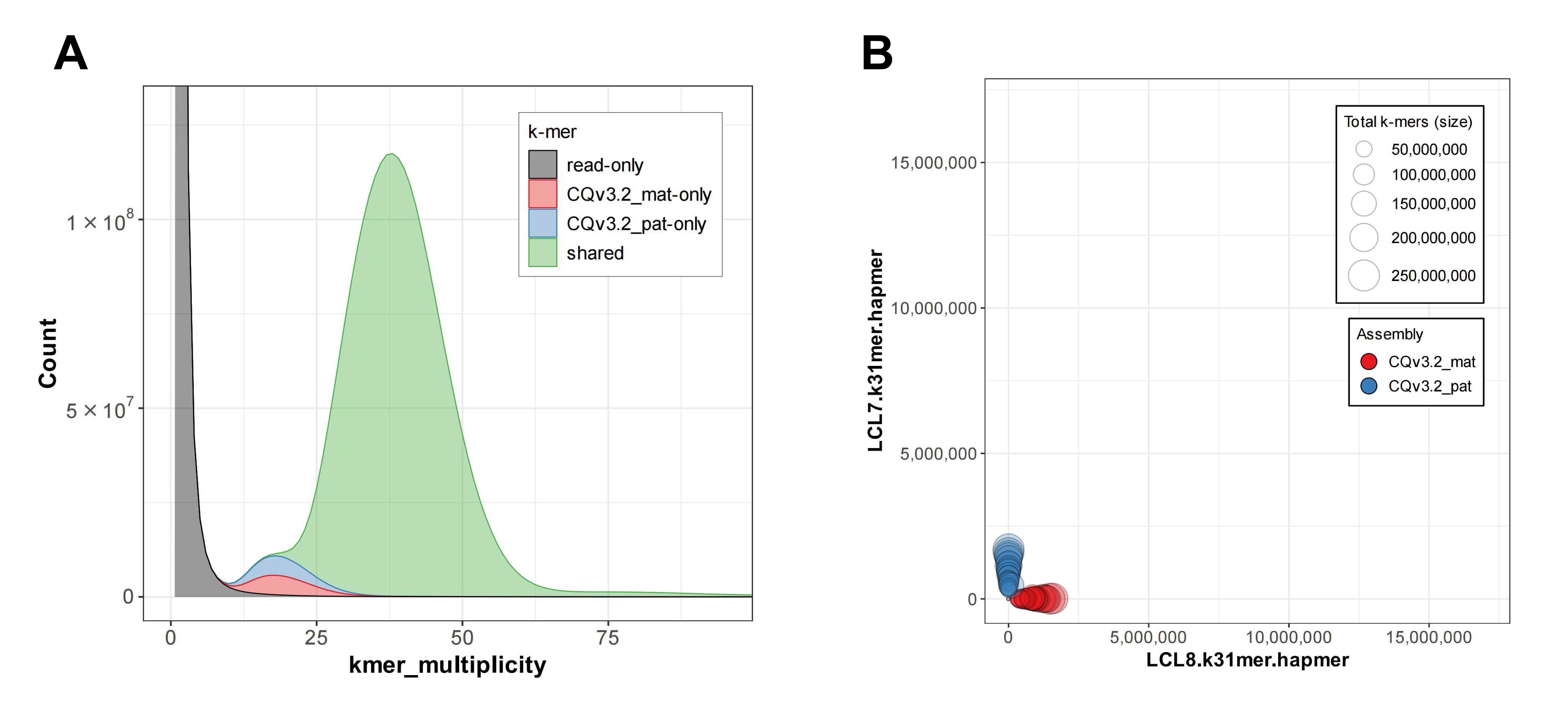

Supplement: qzaf118_Supplementary_Data [file qzaf118_supplementary_data.zip › Figure S4.jpg]

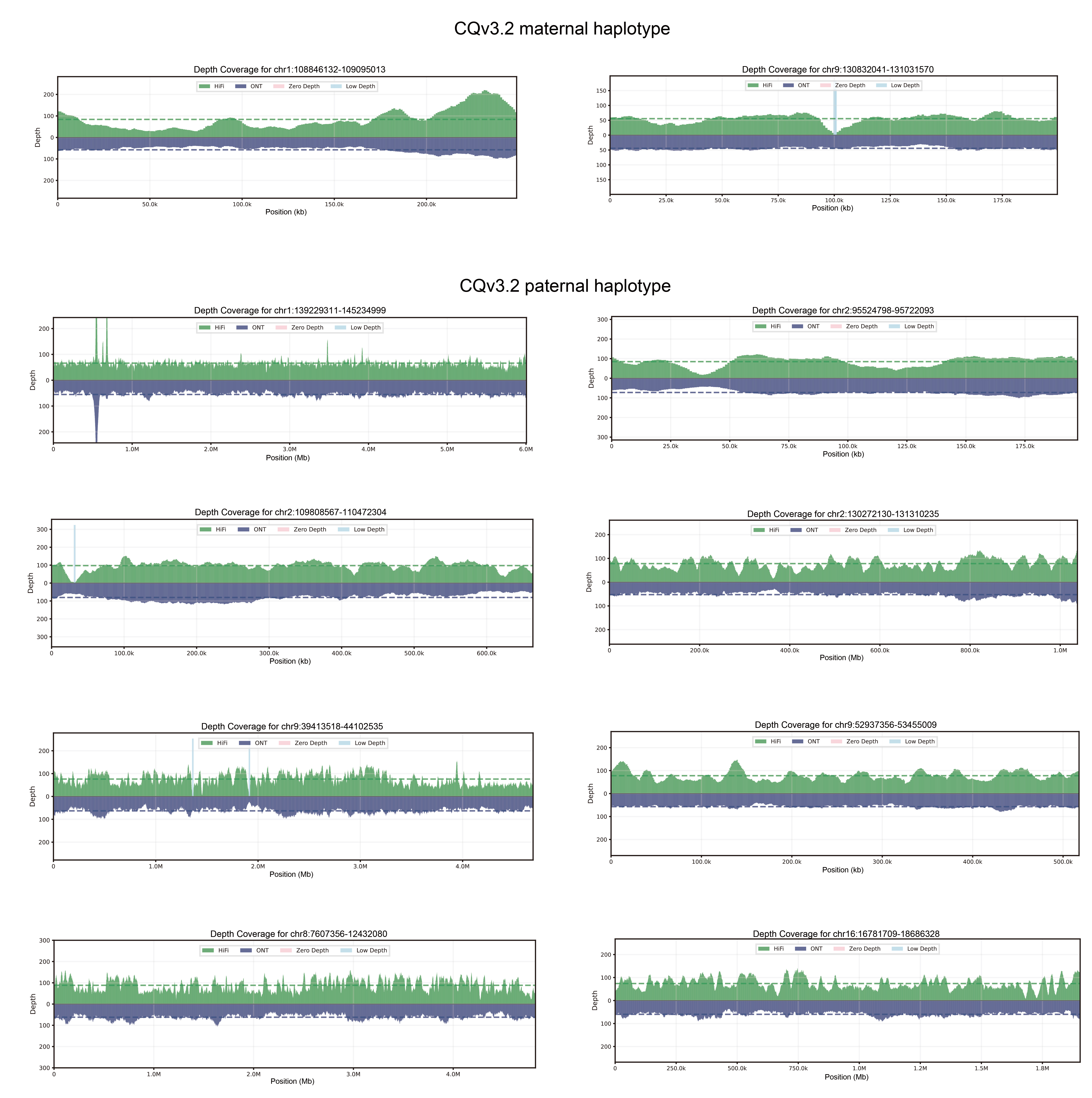

Supplement: qzaf118_Supplementary_Data [file qzaf118_supplementary_data.zip › Figure S7.jpg]

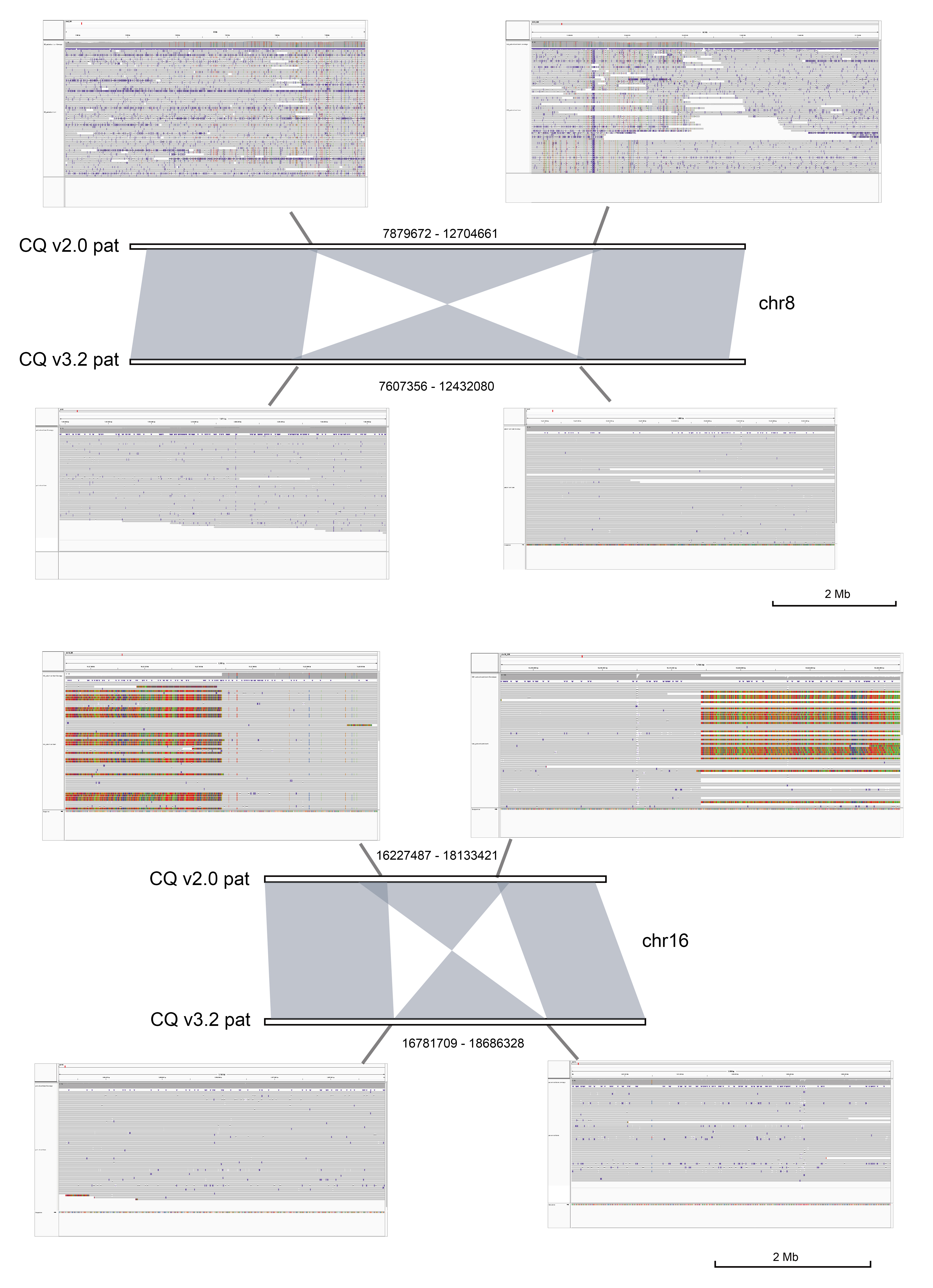

Supplement: qzaf118_Supplementary_Data [file qzaf118_supplementary_data.zip › Figure S8.jpg]

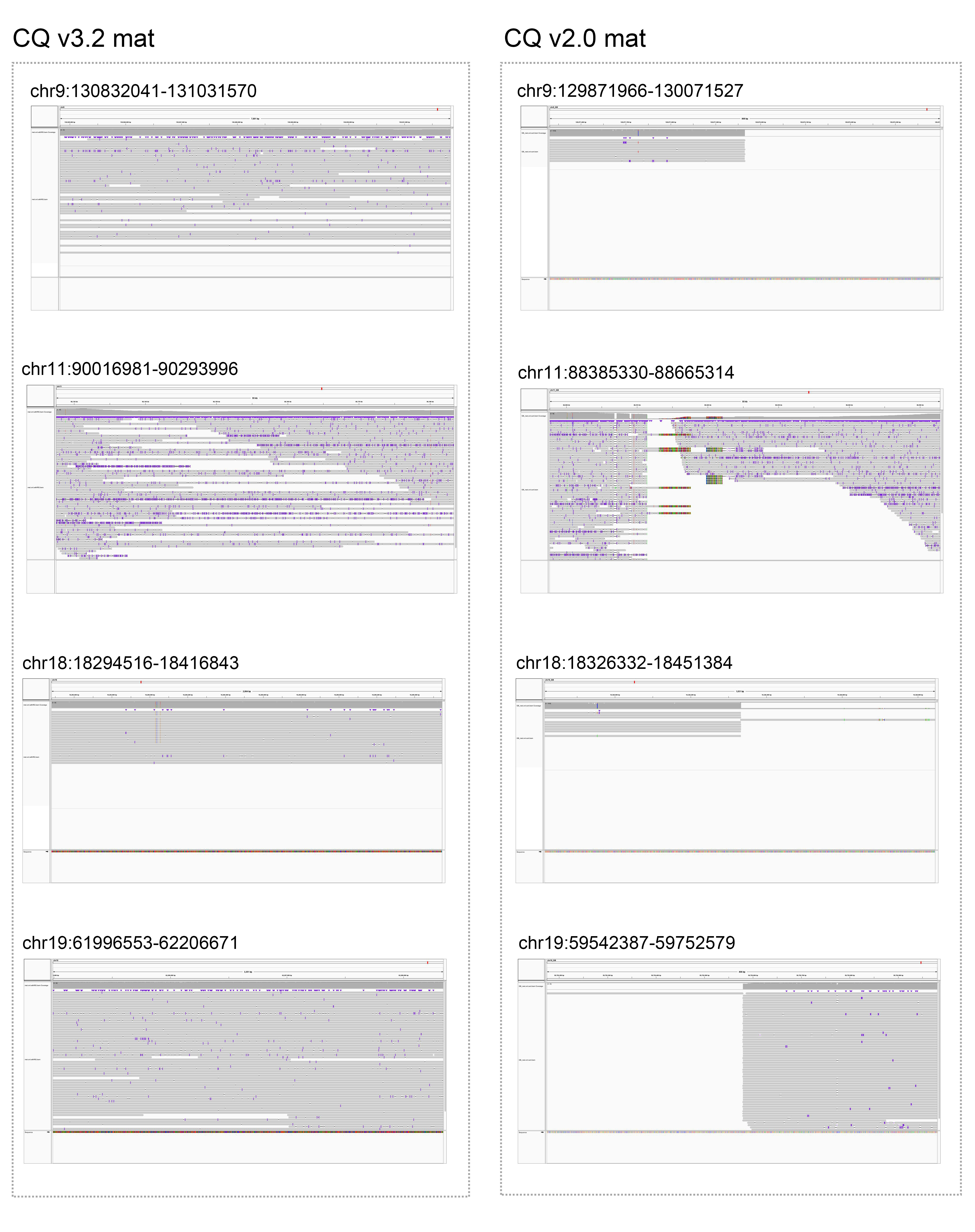

Supplement: qzaf118_Supplementary_Data [file qzaf118_supplementary_data.zip › Figure S9.jpg]

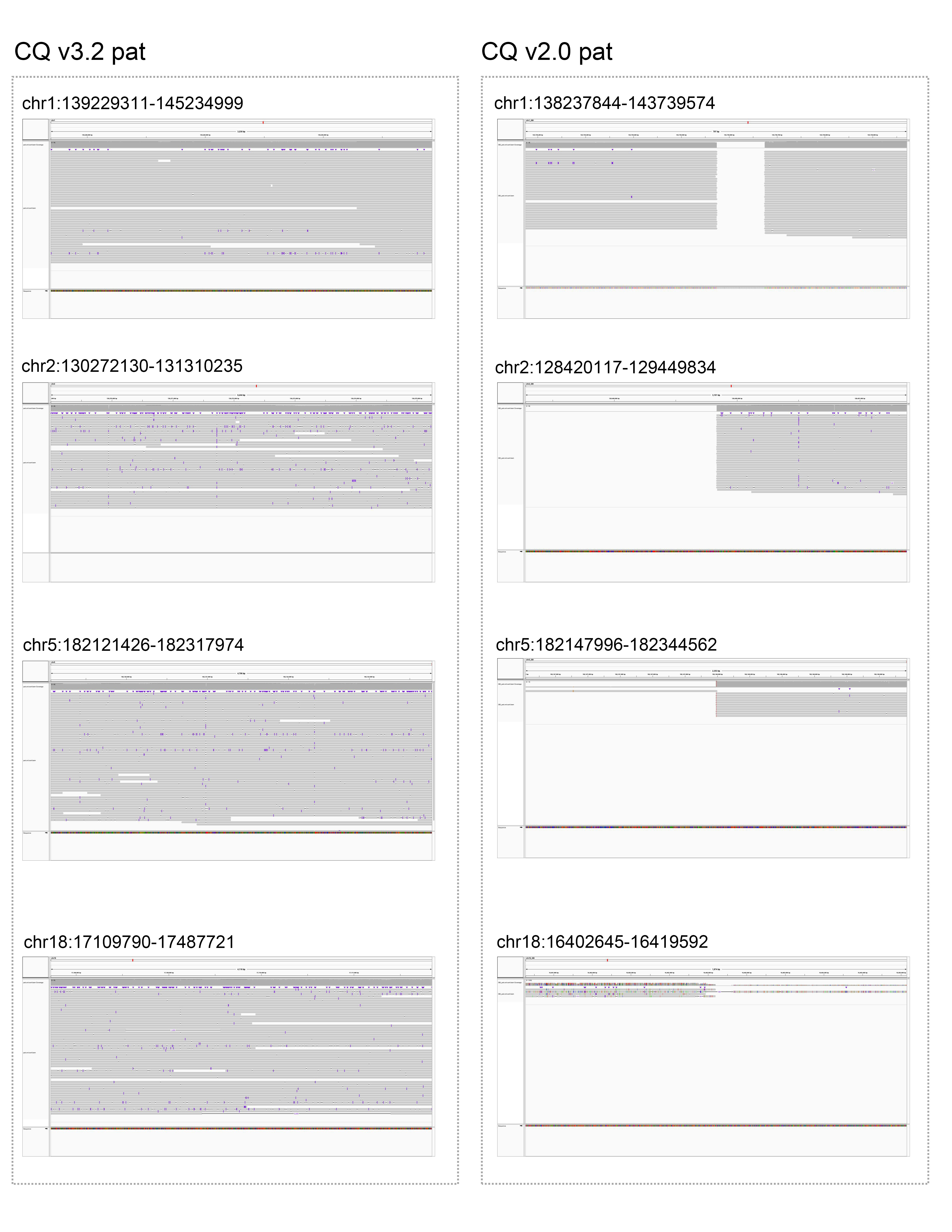

Supplement: qzaf118_Supplementary_Data [file qzaf118_supplementary_data.zip › Figure S10.jpg]

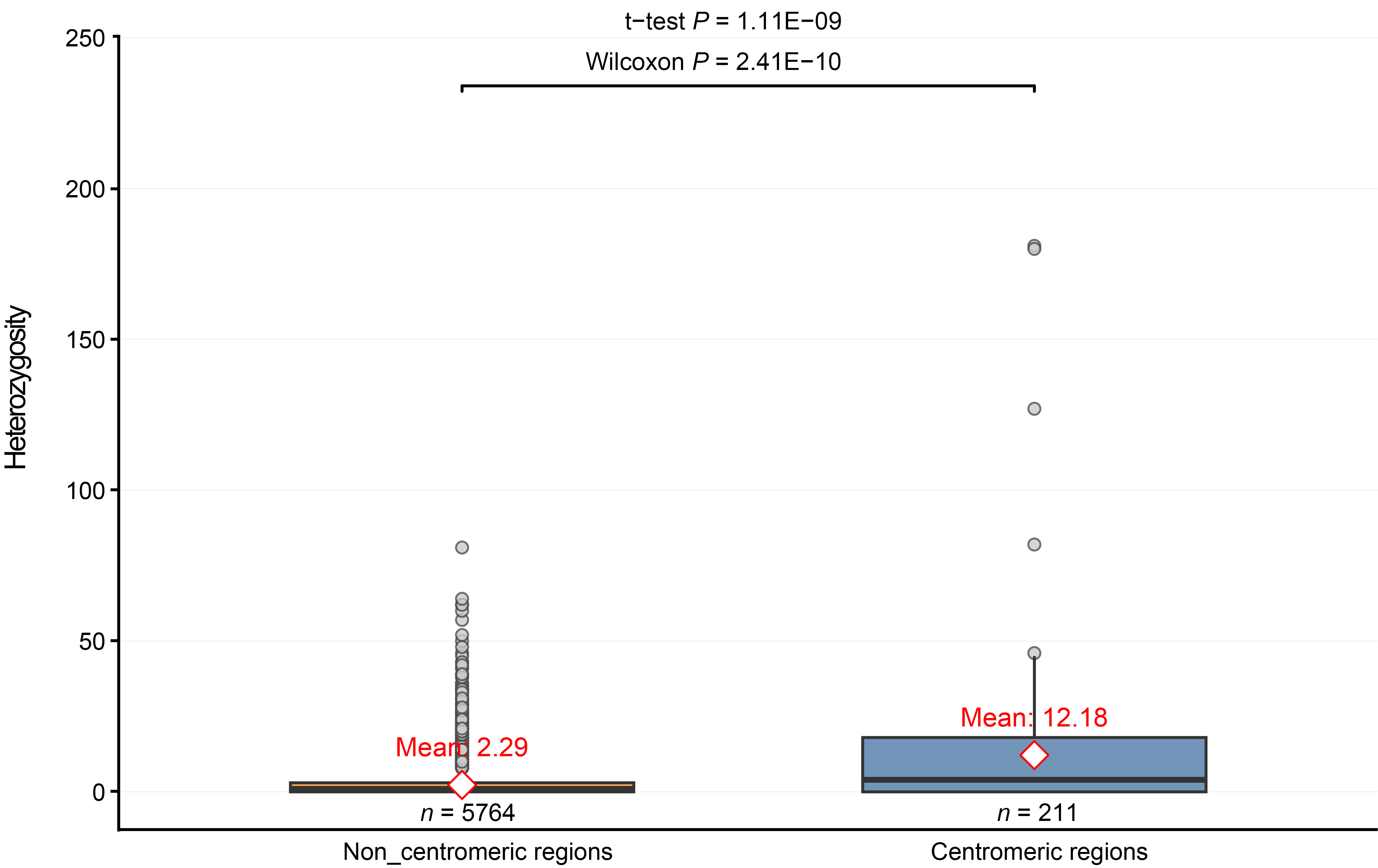

Supplement: qzaf118_Supplementary_Data [file qzaf118_supplementary_data.zip › Figure S11.jpg]

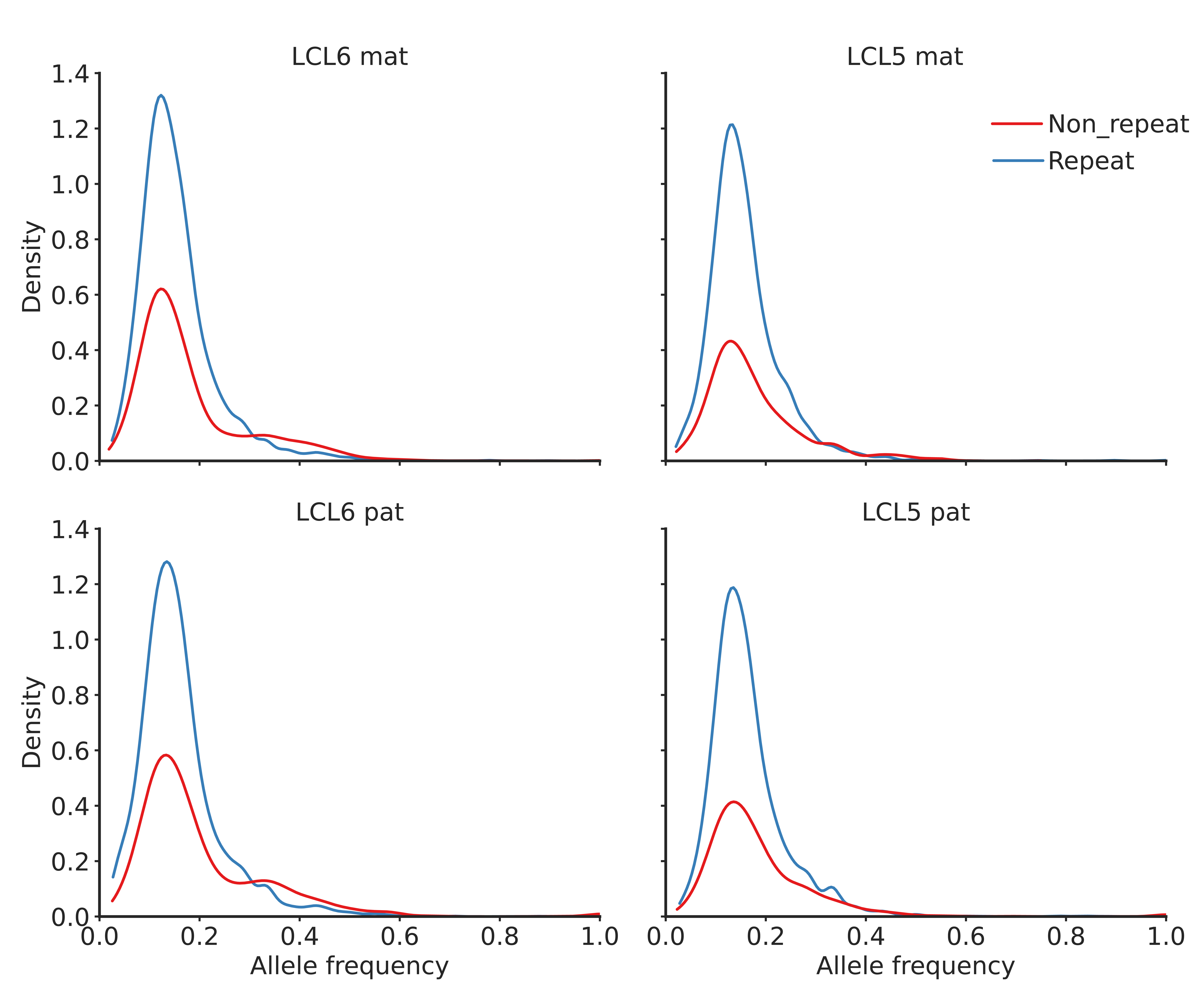

Supplement: qzaf118_Supplementary_Data [file qzaf118_supplementary_data.zip › Figure S12.jpg]

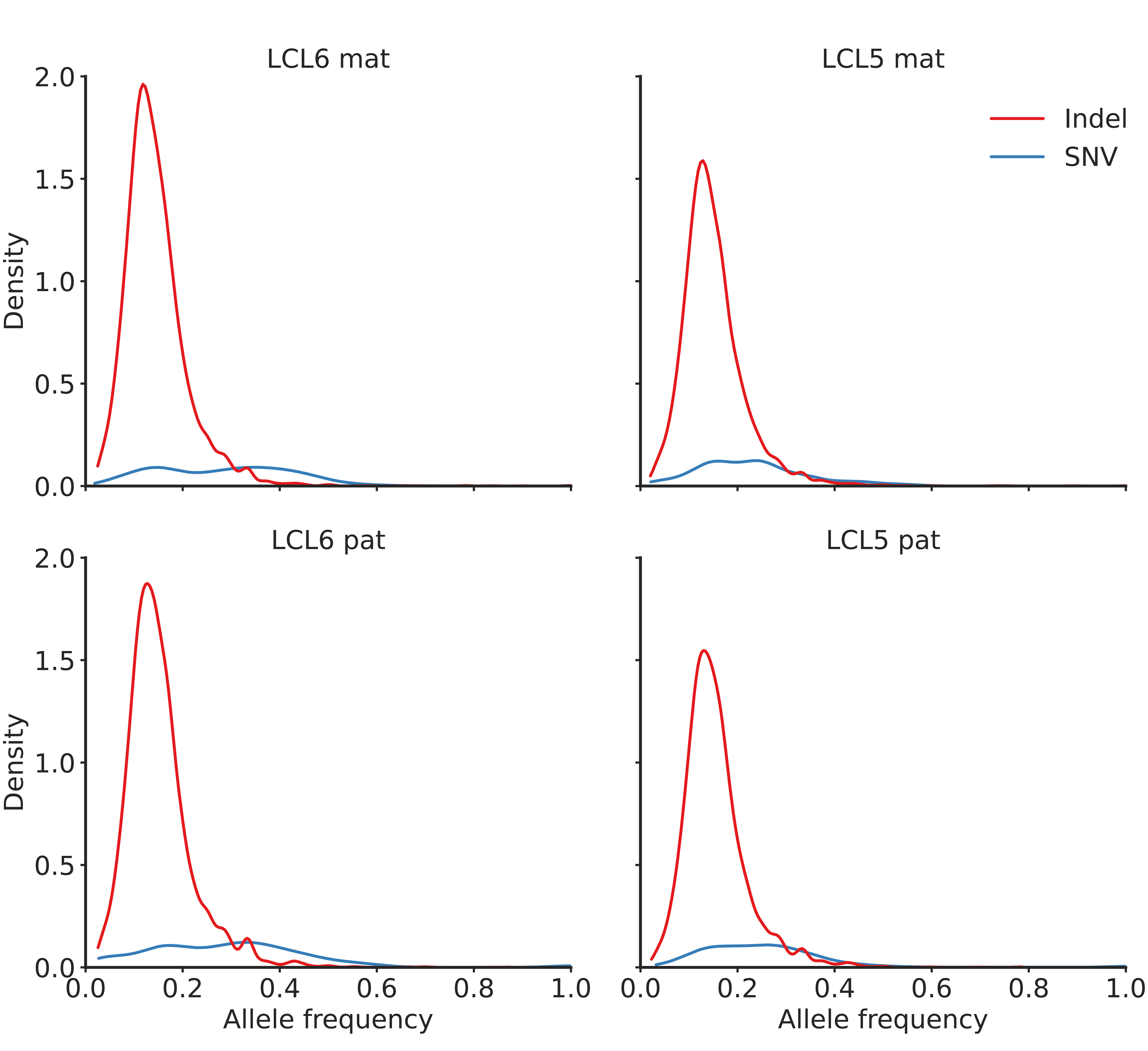

Supplement: qzaf118_Supplementary_Data [file qzaf118_supplementary_data.zip › Figure S13.jpg]

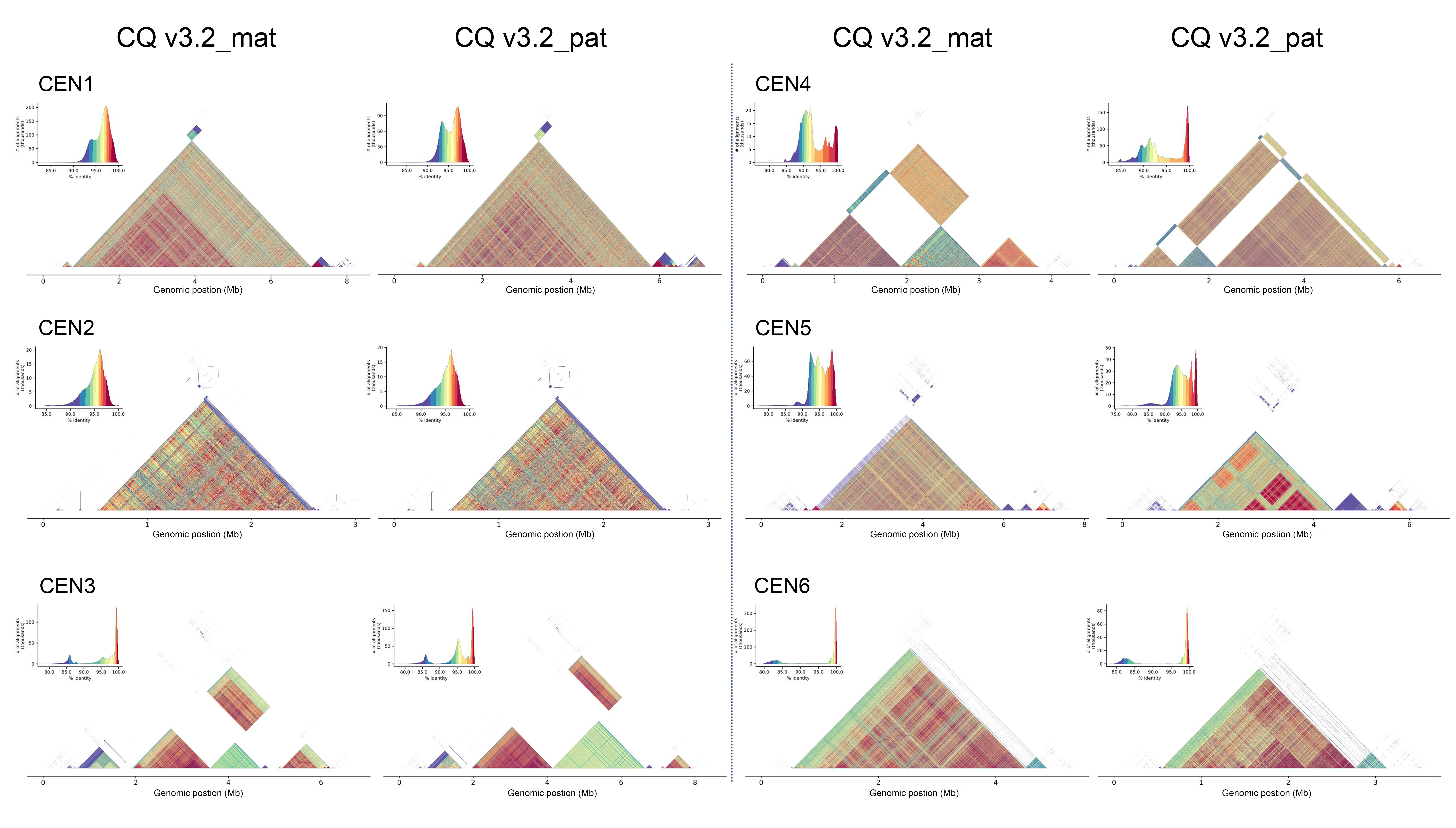

Supplement: qzaf118_Supplementary_Data [file qzaf118_supplementary_data.zip › Figure S14.jpg]

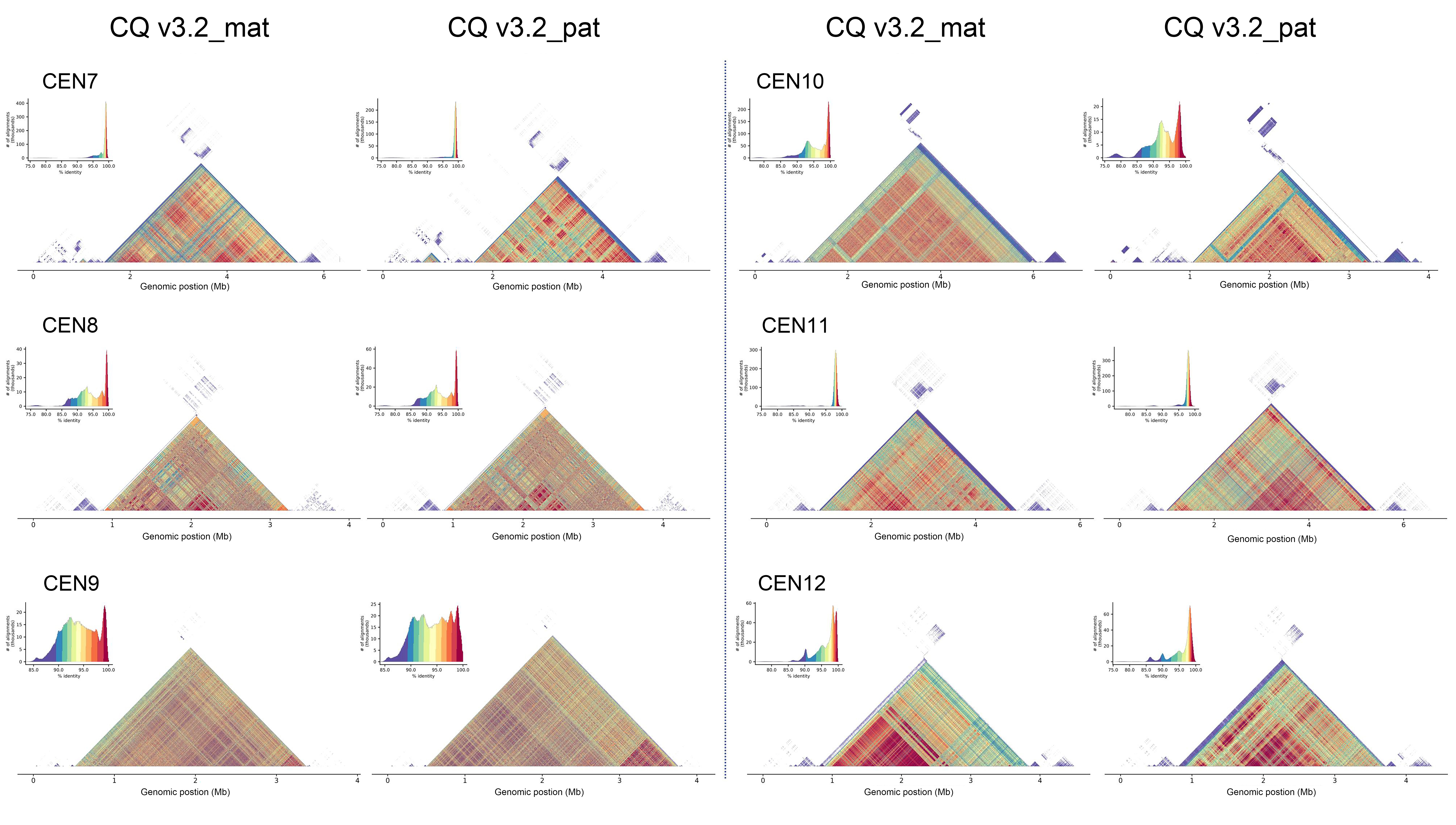

Supplement: qzaf118_Supplementary_Data [file qzaf118_supplementary_data.zip › Figure S15.jpg]

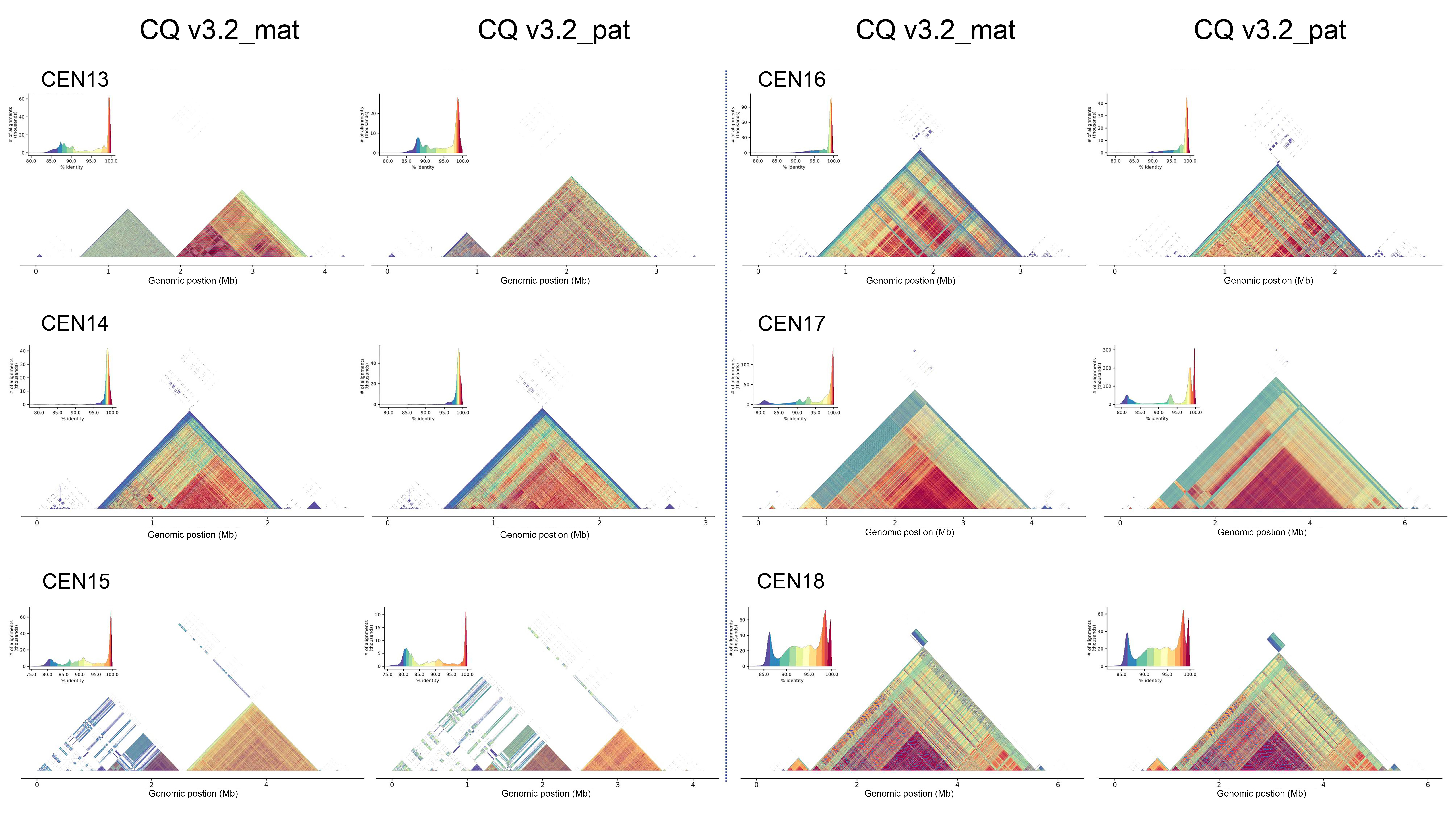

Supplement: qzaf118_Supplementary_Data [file qzaf118_supplementary_data.zip › Figure S16.jpg]

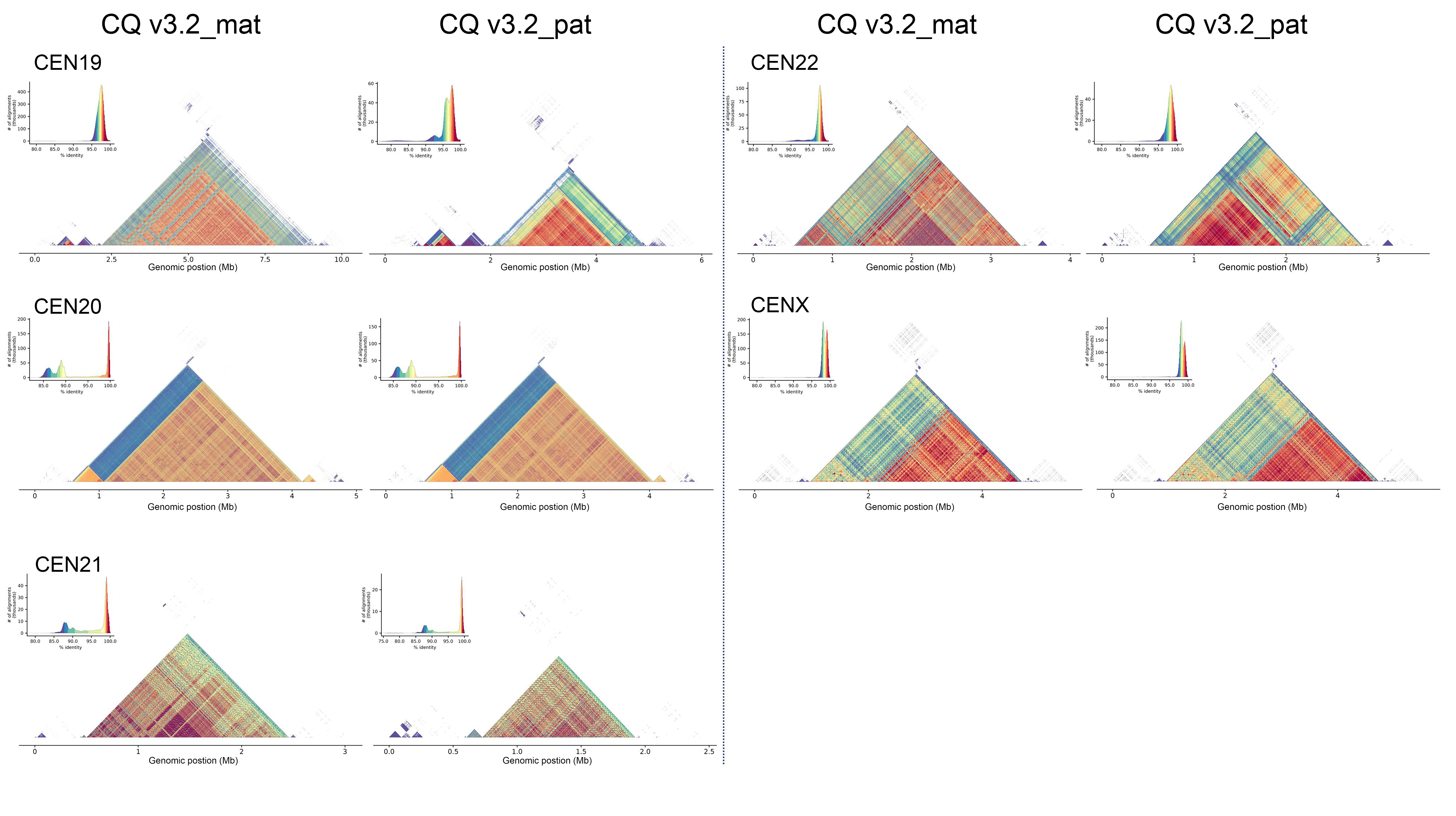

Supplement: qzaf118_Supplementary_Data [file qzaf118_supplementary_data.zip › Figure S17.jpg]
